# Development and external validation of an interpretable deep learning model for early immune-related adverse events in hepatocellular carcinoma

**DOI:** 10.3389/fimmu.2026.1886946

**Published:** 2026-09-21

**Authors:** Hongming Pan, Chunyan Zhao, Hao Sun, Han Zhao, Guhang Tang, Xianfeng Du, Taiyu Xia

**Affiliations:** 1 Department of Oncology, The Affiliated Dazu Hospital of Chongqing Medical University, Chongqing, China; 2 Department of Gastrointestinal Surgery, Harbin Medical University Cancer Hospital, Harbin, Heilongjiang, China; 3 Department of Breast Surgery, Sixth Affiliated Hospital of Harbin Medical University, Harbin, Heilongjiang, China

**Keywords:** hepatocellular carcinoma, immune checkpoint inhibitors, immune-related adverse events, interpretable deep learning, risk prediction

## Abstract

**Objective:**

To develop and externally validate an interpretable deep learning model for pre-treatment prediction of early clinically significant immune-related adverse events in patients with hepatocellular carcinoma receiving immune checkpoint inhibitor–based therapy.

**Methods:**

We conducted a multicenter retrospective cohort study of patients with hepatocellular carcinoma receiving immune checkpoint inhibitor–based therapy, with atezolizumab plus bevacizumab serving as the representative regimen in this cohort. The study population was divided into a training cohort and an independent external validation cohort. Early immune-related adverse events were defined as clinically significant events occurring within three months after treatment initiation. Five prediction models were constructed using baseline clinical and circulating immunological variables obtained prior to treatment, including TabNet, logistic regression, random forest, extreme gradient boosting, and support vector machine models. Model performance was evaluated using discrimination, calibration, and clinical utility metrics, and model interpretability was assessed using feature attribution analyses.

**Results:**

Among all models, the TabNet model showed the most favorable overall performance profile, considering discrimination, calibration, clinical utility, and external validation performance. Calibration analysis showed good agreement between predicted risks and observed event rates, while decision curve analysis and clinical impact assessment indicated meaningful clinical utility across a range of threshold probabilities. Interpretability analyses identified the CD4 to CD8 ratio, serum immunoglobulin G level, albumin, platelet count, and the proportion of CD19-positive B cells as key contributors to model-predicted early immune-related adverse event risk. Risk-stratified feature clustering further suggested that immune-related features and host functional reserve may represent complementary dimensions associated with model-predicted early immune-related toxicity risk.

**Conclusions:**

An interpretable deep learning model was developed and externally validated for pre-treatment prediction of early clinically significant immune-related adverse events in hepatocellular carcinoma patients receiving immune checkpoint inhibitors.

## 1 Introduction

Immune checkpoint inhibitors (ICIs) have fundamentally transformed the therapeutic landscape of hepatocellular carcinoma (HCC), particularly for patients with advanced-stage disease (1). The combination of atezolizumab plus bevacizumab has demonstrated superior survival benefits compared with previous standard therapies and is now widely adopted as first-line treatment (2, 3). However, alongside these therapeutic gains, immune-related adverse events (irAEs) have emerged as a major clinical challenge. IrAEs can affect multiple organ systems and frequently lead to treatment interruption, systemic immunosuppression, or permanent discontinuation of ICIs, thereby compromising treatment continuity, patient safety, and real-world effectiveness (4–6). As the use of ICIs continues to expand in HCC, identifying patients at increased risk of clinically significant irAEs has become an important unmet clinical need.

IrAEs are increasingly recognized as manifestations of systemic immune dysregulation induced by checkpoint blockade (7). Susceptibility to irAEs has been associated with baseline host immune status, inflammatory burden, and organ functional reserve prior to treatment initiation (8). Nevertheless, current clinical practice lacks reliable tools for prospective, pre-treatment risk assessment. Most existing studies have focused on post-treatment biomarkers, single clinical variables, or the overall occurrence of irAEs without differentiating between early- and late-onset toxicities (9). Such approaches may limit clinical applicability, particularly during the initial treatment phase when management decisions are most critical.

Importantly, early-onset clinically significant irAEs may represent a biologically and clinically distinct phenotype (10). Events occurring within the first few treatment cycles are more likely to reflect baseline immune predisposition and host susceptibility rather than cumulative immune perturbation induced by prolonged exposure (11). In contrast, late irAEs may be influenced by dynamic immune remodeling and evolving tumor–immune interactions (12). Pooling early and late toxicities into a single composite endpoint may therefore obscure meaningful heterogeneity and reduce the precision of risk stratification. In routine clinical practice, irAEs of grade ≥2 is particularly relevant because they typically require active intervention, such as treatment delay or corticosteroid initiation, and may disrupt early therapeutic continuity.

Recent advances in machine learning provide opportunities to integrate multidimensional clinical and immunological data for individualized risk prediction. Deep learning models designed for tabular data, such as TabNet, enable modeling of nonlinear feature interactions while maintaining interpretability through attention-based feature selection (13). However, externally validated and interpretable deep learning models specifically targeting early clinically significant irAEs in HCC remain limited. In this study, we developed and externally validated an interpretable deep learning model to predict the risk of early-onset clinically significant irAEs (grade ≥2) in patients with HCC receiving atezolizumab plus bevacizumab as a representative ICI-based combination regimen. By integrating routinely available clinical variables with baseline immunological markers obtained prior to treatment initiation, we aimed to establish a pre-treatment risk prediction framework that balances predictive accuracy, generalizability, and interpretability, and to elucidate baseline immune features associated with early immune-related toxicity.

## 2 Patients and methods

### 2.1 Patients

This study was designed as a multicenter retrospective cohort study. We initially screened 741 patients with HCC who received treatment at participating centers between May 2020 and December 2023. Patients were allocated to the training or external validation source cohorts according to the hospital of treatment. The training source cohort included 518 patients from Harbin Medical University Cancer Hospital. After applying the predefined eligibility criteria, 453 patients were included as the final training cohort, with 141 early clinically significant irAE events. The external validation source cohort included 223 patients from two independent centers, including the Sixth Affiliated Hospital of Harbin Medical University (n = 37) and the People’s Hospital of Dazu District, Chongqing (n = 186). After screening, 201 patients were included in the final external validation cohort, including 31 patients from the Sixth Affiliated Hospital of Harbin Medical University and 170 patients from the People’s Hospital of Dazu District, Chongqing, contributing 7 and 52 early clinically significant irAE events, respectively. The training and external validation cohorts were separated at the hospital level, and no patient overlap existed between the two cohorts. The same inclusion and exclusion criteria were applied across participating centers. The detailed patient screening and exclusion process is shown in **Figure 1**; specifically, 13 patients were excluded because of missing prespecified key baseline variables, including 9 from the training source cohort and 4 from the external validation source cohort.

**Figure 1:**
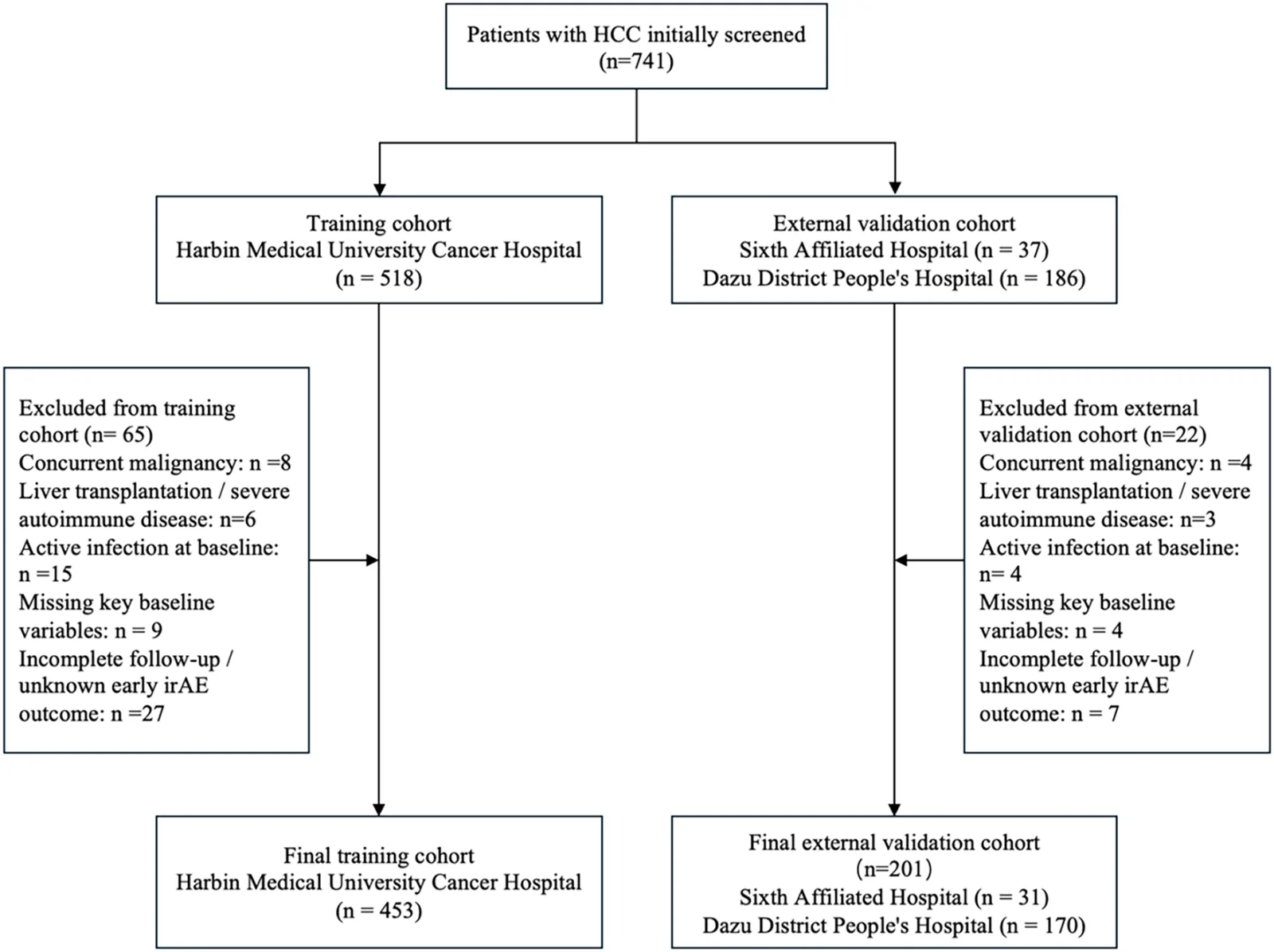
Patient selection flowchart.

The diagnosis of HCC was established in accordance with the guidelines of the Chinese Society of Clinical Oncology (CSCO), based on histopathological examination or noninvasive imaging criteria using contrast-enhanced computed tomography (CT) or magnetic resonance imaging (MRI) (14). To ensure population homogeneity and data quality for model development, uniform eligibility criteria were applied across participating centers.

The inclusion criteria were as follows: (1) age ≥18 years; (2) confirmed diagnosis of HCC; and (3) receipt of atezolizumab plus bevacizumab (Atezo+Bev) therapy with at least one complete treatment cycle. Patients meeting any of the following criteria were excluded: (1) presence of another concurrent primary malignancy; (2) history of liver transplantation or severe autoimmune disease; (3) absence of prespecified key pretreatment clinical, laboratory, serological, or immunological variables included in the predefined predictor set; (4) incomplete follow-up or unknown early irAE outcome within the prespecified 3-month observation window; and (5) evidence of active infection at baseline requiring systemic antibiotic therapy, which could confound baseline inflammatory or immunological biomarker measurements.

This multicenter retrospective study was reviewed and approved by the Ethics Committee of Harbin Medical University Cancer Hospital (Approval No. ALTN-AK105-III-06), the Ethics Committee of the Sixth Affiliated Hospital of Harbin Medical University (Approval No. LC2024-052), and the Ethics Committee of the Affiliated Dazu Hospital of Chongqing Medical University (Approval No. 2026 Scientific Ethics Review No. 038). All procedures were conducted in accordance with the Declaration of Helsinki. Written informed consent for the use of clinical data for research purposes was obtained from all participants.

### 2.2 Treatment regimen and definition of irAEs

All patients received the atezolizumab plus bevacizumab (Atezo+Bev) regimen, a widely used immune checkpoint inhibitor–based combination regimen for HCC. Atezolizumab was administered intravenously at a fixed dose of 1200 mg, and bevacizumab was administered intravenously at a dose of 15 mg/kg, with each treatment cycle repeated every 3 weeks. To ensure treatment exposure, only patients who received at least one complete treatment cycle were included in the analysis. The number of completed Atezo+Bev treatment cycles during the prespecified 3-month observation window was recorded descriptively and was not included as a model predictor. Patients who discontinued treatment because of an irAE were retained in the analysis and classified as having experienced the study outcome. Patients who discontinued treatment because of disease progression or other non-irAE reasons remained eligible if their early irAE status could still be clearly ascertained within the 3-month observation window. Patients with incomplete follow-up or an indeterminate early irAE outcome were excluded rather than classified as non-irAE cases.

Immune-related adverse events were defined as adverse events with a suspected immune-mediated etiology occurring after initiation of ICIs therapy. All irAEs were graded by treating physicians according to the Common Terminology Criteria for Adverse Events (CTCAE), version 5.0, and events of grade ≥2 were considered clinically significant. The ascertainment of irAEs was based on systematic review of electronic medical records, including outpatient and inpatient clinical notes, laboratory test results, imaging findings, and relevant specialist consultation records. During the original outcome assessment process, suspected irAEs were first identified based on clinical diagnosis and medical records and were then reviewed by a panel of two to three experienced clinicians according to a unified CTCAE v5.0-based adjudication standard. Final classification was determined by consensus discussion. For each suspected event, adjudication considered symptom onset, temporal relationship with Atezo+Bev therapy, laboratory trends, imaging findings, microbiological evidence, tumor progression status, concomitant medications, and relevant specialist consultation records. In patients with HCC, particular attention was paid to distinguishing immune-related toxicity from hepatic decompensation, infection, disease progression, bevacizumab-related adverse events, and fluctuations in underlying liver disease. Events with a more plausible non-immune-related etiology were not classified as irAEs.

To enhance clinical interpretability and focus on early immune toxicity that may directly affect treatment monitoring and management, outcome assessment was restricted to a prespecified 3-month observation window following the first ICIs therapy. The follow-up cutoff date was March 21, 2024. Patients were followed from treatment initiation until the occurrence of the first qualifying irAE, death, the last clinical contact, or completion of the 3-month observation period, whichever occurred first. Patients were classified into the irAE and non-irAE groups based on the occurrence of at least one CTCAE grade ≥2 irAE within this early observation window. For descriptive profiling of early clinically significant irAEs, organ-system category, CTCAE grade, time to onset, glucocorticoid use, treatment interruption, and permanent discontinuation were also extracted from medical records. These irAE-related characteristics were used exclusively for descriptive clinical profiling and were not included as model predictors, because they were determined after treatment initiation; the prespecified prediction outcome was the occurrence of at least one grade ≥2 irAE within the 3-month observation window.

### 2.3 Candidate predictors and baseline assessment

Candidate predictors were prespecified based on clinical relevance, biological plausibility, and availability in routine clinical practice, with the aim of enabling true pre-treatment risk prediction. The required baseline variables were prespecified before model development and were not selected retrospectively according to the performance or requirements of any specific algorithm. Given the immune-mediated nature of irAEs, candidate variables were defined to comprehensively capture baseline host immune status in addition to conventional clinical and laboratory indicators. Only variables obtained prior to the initiation of ICIs therapy were considered for model development. No univariate pre-screening was performed before model development because the candidate predictors had been prespecified on clinical and biological grounds, and variables with limited marginal associations could still contribute to multivariable prediction through nonlinear relationships or interactions with other features.

A total of 51 pretreatment candidate predictors were prespecified for model development. Clinical variables included age, body mass index (BMI), sex, smoking status, drinking status, Child–Pugh class, ABO blood type, previous surgery, tumor number, tumor size, liver cirrhosis, and BCLC stage. Immunological variables included serum immunoglobulin A (IgA), immunoglobulin G (IgG), and immunoglobulin M (IgM); the proportions of CD3+, CD4+, and CD8+ T cells; the CD4+/CD8+ ratio; CD3+CD4+CD8+ cells; CD19+ B cells; CD3−CD16+CD56+ cells; and CD3+CD16+CD56+ cells. Laboratory variables included alanine aminotransferase (ALT), aspartate aminotransferase (AST), γ-glutamyl transferase (γ-GGT), lactate dehydrogenase (LDH), total bilirubin (TBIL), direct bilirubin (DBIL), indirect bilirubin (IDBIL), total protein (TP), albumin (ALB), globulin (GLOB), albumin-to-globulin ratio (A/G), prealbumin (PALB), blood urea nitrogen (BUN), creatinine (CREA), uric acid (UA), alkaline phosphatase (ALP), glucose (Glu), white blood cell count (WBC), neutrophil count (NEU), lymphocyte count (LYM), monocyte count (MON), red blood cell count (RBC), hemoglobin (HGB), hematocrit (HCT), and platelet count (PLT). Coagulation-related variables included international normalized ratio (INR), fibrinogen (Fbg), and D-dimer.

All baseline assessments were performed before the first administration of ICIs. For laboratory measurements, baseline values were defined as the most recent results obtained within 7 days prior to ICIs therapy. When multiple measurements were available within this time window, the value closest to treatment initiation was selected. All baseline data were extracted from electronic medical records and cross-checked for internal consistency and clinical plausibility.

Continuous variables were retained in their original scale to preserve quantitative information and were standardized as required for model input. Categorical variables were encoded using appropriate binary or ordinal representations. To prevent information leakage, no post-treatment variables or time-dependent features were included in the analysis. To ensure data quality and model comparability across algorithms, only patients with complete baseline data for the prespecified candidate predictors were included in the final analysis in accordance with the predefined complete-case strategy. As a result, no missing data imputation was performed in this study. Detailed definitions and distributions of the candidate predictors are provided in **Supplementary Table 3**. With 141 early clinically significant irAE events in the training cohort, the crude events-per-candidate-predictor ratio was 2.76.

### 2.4 Model development and validation

Five prediction models, including TabNet, logistic regression, random forest, extreme gradient boosting (XGBoost), and support vector machine, were developed in parallel using the same set of baseline predictors. All models were trained exclusively in the training cohort and evaluated in an independent external validation cohort, with no information from the validation cohort used during model training or hyperparameter optimization to prevent information leakage. For logistic regression, multicollinearity among candidate variables was assessed using tolerance and variance inflation factor (VIF) to ensure model stability. Hyperparameter tuning was performed exclusively within the training cohort using stratified 5-fold cross-validation, with AUC as the tuning metric. During cross-validation, all preprocessing procedures, including continuous-variable standardization and categorical-variable encoding, were fitted exclusively within each training fold and then applied to the corresponding held-out fold. After hyperparameter selection, preprocessing parameters were re-estimated using the entire training cohort and applied unchanged to the external validation cohort. No preprocessing parameters were derived from the external validation data. Class imbalance was addressed using class-weighted learning or model-specific weighting strategies where applicable, and early stopping was applied for TabNet and XGBoost. The hyperparameter tuning ranges and final selected parameters of all models are provided in **Supplementary Table 1**. The optimized models were subsequently fixed and applied to the external validation cohort without further adjustment.

To further assess potential overfitting and quantify optimism associated with model development, internal validation of the final TabNet model was performed using 500 bootstrap resamples of the training cohort. In each bootstrap iteration, preprocessing parameters were estimated from the bootstrap sample, and the model was refitted using the final hyperparameters selected exclusively within the original training cohort. Performance was evaluated in both the bootstrap sample and the original training cohort. For each metric, optimism was defined as the mean signed difference between performance in the bootstrap sample and performance of the same bootstrap-fitted model in the original training cohort. Optimism-corrected performance was calculated by subtracting the estimated optimism from the apparent performance. The evaluated measures included AUC, Brier score, calibration intercept, and calibration slope.

As an additional sensitivity analysis addressing the relatively limited number of outcome events in relation to the original predictor set, a reduced-feature TabNet model was developed using the 10 predictors with the highest SHAP importance identified exclusively within the training cohort. The reduced-feature model was trained and optimized using the same model-development framework as the primary TabNet model and was subsequently applied without modification to the independent external validation cohort. No information from the external validation cohort was used for feature selection, model training, or hyperparameter optimization. Discrimination and calibration of the reduced-feature model were evaluated using AUC, Brier score, calibration intercept, calibration slope, ROC curves, and calibration curves and were compared with those of the full-feature TabNet model.

Model discrimination was evaluated using the concordance index (C-index) and receiver operating characteristic (ROC) curves, with the area under the curve (AUC) calculated for both the training and validation cohorts. In addition to AUC/C-index, threshold-based classification metrics, including sensitivity, specificity, positive predictive value, negative predictive value, and F1 score, were calculated for each model. For each model, the optimal cut-off was determined in the training cohort using the Youden index and then applied to the external validation cohort without further adjustment. Brier score, calibration intercept, and calibration slope were calculated to further evaluate the accuracy and calibration of predicted probabilities. To compare discrimination between TabNet and comparator models, bootstrap resampling was performed in the external validation cohort. Differences in AUC were estimated by resampling patients with replacement for 5,000 iterations, and 95% confidence intervals were calculated using the percentile method. Two-sided P values were derived from the bootstrap distribution of AUC differences. Model calibration was assessed using calibration curves comparing predicted and observed event rates. Clinical utility was evaluated using decision curve analysis (DCA), which estimates the net benefit of model-assisted decision-making across a range of clinically relevant threshold probabilities. In addition, precision–recall (PR) curves were generated to further assess performance under class imbalance. Clinical impact curves (CICs) were constructed to visualize the estimated number of patients classified as high risk and the corresponding number of true positive events across different threshold probabilities, thereby illustrating the potential population-level impact of applying the prediction model in clinical practice.

To examine the intrinsic interpretability of the final TabNet model, attention-based feature masks were extracted to visualize global and sample-level feature-selection patterns. SHapley Additive exPlanations (SHAP) were then applied as a complementary *post hoc* interpretation method to evaluate the direction and magnitude of feature contributions to individual risk predictions. For risk-stratified SHAP analysis, patients were divided into low-, intermediate-, and high-predicted-risk strata according to tertiles of the predicted probabilities generated by the final TabNet model.

For threshold-based clinical risk stratification, the final TabNet model was used to generate an individualized predicted probability of early clinically significant irAEs for each patient. The optimal cut-off was determined in the training cohort using the Youden index and was then fixed and applied to the external validation cohort without further adjustment. Patients with predicted probabilities equal to or above the cut-off were classified as high risk, whereas those below the cut-off were classified as non-high risk. In the external validation cohort, the observed incidence of early clinically significant irAEs was compared between the high-risk and non-high-risk groups. The association between threshold-defined high-risk status and early irAEs was estimated using odds ratios with 95% confidence intervals. Threshold-based performance metrics, including sensitivity, specificity, positive predictive value, negative predictive value, accuracy, and F1 score, were calculated to assess the clinical classification performance of the risk threshold.

### 2.5 Statistical analysis

Descriptive statistics were used to summarize baseline characteristics of the study population. Continuous variables were expressed as mean ± standard deviation or median (interquartile range), as appropriate based on data distribution, while categorical variables were presented as counts and percentages. Group comparisons were performed using the Student’s *t* test or Mann–Whitney *U* test for continuous variables and the χ² test or Fisher’s exact test for categorical variables, as appropriate. To assess potential inter-center heterogeneity within the external validation cohort, baseline characteristics were compared across the contributing centers using standardized mean differences (SMDs). Model development and evaluation were conducted strictly according to the predefined analytical plan. All analyses were performed separately in the training cohort and the external validation cohort to avoid information leakage. Model discrimination, calibration, and clinical utility were assessed using prespecified performance metrics as described above. No *post hoc* modifications to model structure or evaluation criteria were made based on validation results. All statistical analyses and model development procedures were implemented using Python (version 3.14) and R (version 4.3.1), with relevant machine learning and statistical packages applied as appropriate. All tests were two-sided, and a *P* value < 0.05 was considered statistically significant unless otherwise specified.

## 3 Results

### 3.1 Patients characteristics

A total of 654 patients were included in the overall study population. The mean age was 57.14 ± 10.97 years, and most patients were male. The mean body mass index (BMI) was 24.09 ± 4.43 kg/m², indicating a predominantly normal to mildly overweight population. With respect to disease characteristics, more than half of the patients had advanced disease, with BCLC stage C accounting for approximately 57% of cases, while the remaining patients were classified as BCLC stage A or B. During the prespecified 3-month observation window, 29 (4.4%), 180 (27.5%), 311 (47.6%), and 134 (20.5%) patients received 1, 2, 3, and 4 complete Atezo+Bev treatment cycles, respectively. Early immune-related adverse events occurred in approximately 30.6% of patients. Among the 200 patients who developed early clinically significant irAEs, hepatic toxicity was the most common category (68/200, 34.0%), followed by cutaneous toxicity (41/200, 20.5%) and endocrine toxicity (34/200, 17.0%). Most events were grade 2 (158/200, 79.0%), whereas grade 3 and grade 4 events accounted for 19.0% and 2.0%, respectively, with a median time to onset of 42 days. Overall, 82 patients (41.0%) received glucocorticoid treatment, 53 (26.5%) experienced treatment interruption, and 12 (6.0%) permanently discontinued treatment because of irAEs. Notably, pneumonitis was less frequent but was associated with relatively high rates of glucocorticoid use, treatment interruption, and permanent discontinuation. These descriptive data characterize the clinical spectrum, severity, and management burden of early irAEs in the study population (**Supplementary Table 2**). Compared with patients without early irAEs, those who developed early irAEs were older (*P = 0.021*), more likely to have underlying liver cirrhosis (*P = 0.009*), and more likely to have tumors smaller than 5 cm (*P = 0.006*). Other baseline characteristics showed no statistically significant differences between patients with and without early irAEs (all *P > 0.05*; **Table 1**).

**Table 1:** Patients characteristics.

	Early irAE (grade ≥2)		
Items	Yes (n = 200)	No (n = 454)	*P*
Age (years), mean (SD)	58.6 (10.4)	56.5 (11.2)	0.021
BMI (Kg/m^2^), mean (SD)	24.3 (4.5)	24.0 (4.4)	0.432
Sex			0.223
Male	168 (84.0%)	363 (79.9%)	
Female	32 (16.0%)	91 (20.1%)	
Smoking			0.077
Yes	53 (26.5%)	92 (20.3%)	
No	147 (73.5%)	362 (79.7%)	
Drinking			0.505
Yes	28 (14.0%)	55 (12.1%)	
No	172 (86.0%)	399 (87.9%)	
Child pugh class			0.097
A	139 (69.5%)	350 (77.1%)	
B	50 (25.0%)	89 (19.6%)	
C	11 (5.5%)	15 (3.3%)	
ABO blood type			0.978
A	53 (26.5%)	122 (26.9%)	
B	64 (32.0%)	139 (30.6%)	
AB	27 (13.5%)	66 (14.5%)	
O	56 (28.0%)	127 (28.0%)	
Surgery			0.108
Yes	74 (37.0%)	139 (30.6%)	
No	126 (63.0%)	315 (69.4%)	
Tumor number			0.724
Single	84 (42.0%)	184 (40.5%)	
Multiple	116 (58.0%)	270 (59.5%)	
Tumor size			0.006
< 5 cm	53 (26.5%)	78 (17.2%)	
≥ 5 cm	147 (73.5%)	376 (82.8%)	
Liver cirrhosis			0.009
Yes	76 (38.0%)	126 (27.8%)	
No	124 (62.0%)	328 (72.2%)	
BCLC stage			0.422
A + B	90 (45.0%)	189 (41.6%)	
C	110 (55.0%)	265 (58.4%)	

### 3.2 Machine learning model development and evaluation

#### 3.2.1 Candidate variables for model development

Baseline candidate variables were prespecified based on clinical relevance and routine availability, including demographic, tumor-related, laboratory, and immunological parameters. All variables were obtained prior to treatment initiation and showed no statistically significant differences between the training cohort and the external validation cohort (*all P > 0.05*), supporting their suitability for subsequent model development and validation (**Supplementary Table 3**). Additional inter-center comparison showed generally limited heterogeneity in baseline clinical, laboratory, and immunological characteristics (**Supplementary Table 4**).

#### 3.2.2 Model performance comparison and generalization assessment

All collected baseline variables were entered into the models without prior univariate filtering. Hyperparameter optimization was applied to all models as described in the Methods section. The predictive performance of five machine learning models was evaluated using the C-index in both the training and external validation cohorts. In the training cohort, XGBoost and TabNet showed the highest numerical discrimination (C-index = 0.85), whereas logistic regression, support vector machine, and random forest showed lower training discrimination. However, differences in generalization performance emerged when model performance was assessed in the external validation cohort (**Figure 2A**). Among all models, TabNet showed the most favorable overall performance profile, with the highest numerical validation C-index (0.78) and a relatively small performance gap between the training and validation cohorts, suggesting stable generalization in this dataset. In contrast, XGBoost exhibited a pronounced decline in validation performance (C-index = 0.69) despite strong discrimination in the training cohort, suggesting potential overfitting. Random forest and support vector machine models showed moderate and relatively stable performance across cohorts, whereas logistic regression showed lower overall discrimination.

**Figure 2:**
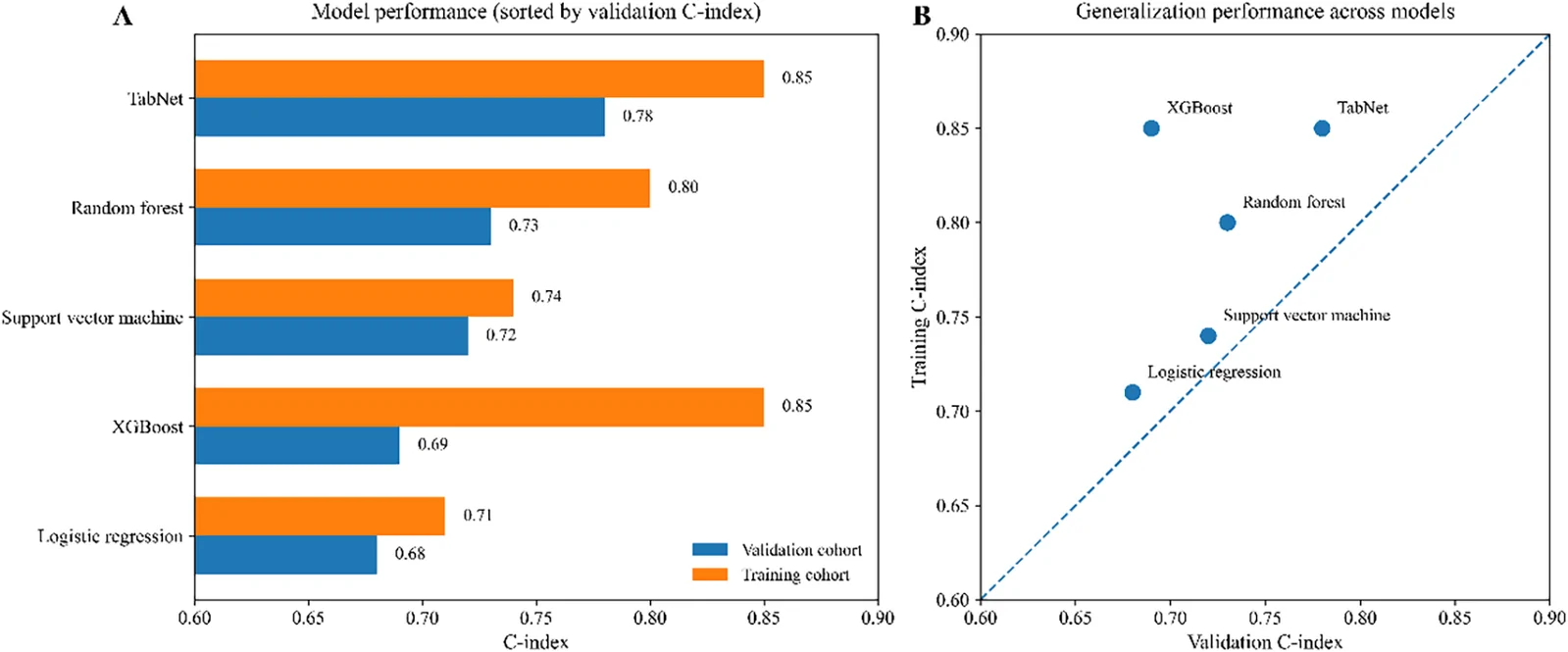
Model performance and generalization. **(A)** Model performance in the training and validation cohorts; **(B)** Model generalization performance.

Complete model performance metrics are summarized in **Supplementary Table 5**. In the external validation cohort, TabNet achieved a higher numerical AUC than random forest, although this difference did not reach statistical significance (ΔAUC = 0.05, 95% CI: −0.015 to 0.112, P = 0.132). TabNet showed significantly higher AUCs than XGBoost, support vector machine, and logistic regression (all P < 0.05; **Supplementary Table 6**). The relationship between training and validation performance further supports these observations, with TabNet located closest to the ideal diagonal line representing balanced generalization, whereas XGBoost deviated markedly from this reference (**Figure 2B**). Taken together, TabNet showed a favorable balance between predictive performance and generalization in this study and was therefore selected as the final modeling approach for subsequent analyses. Bootstrap internal validation demonstrated limited optimism in the final TabNet model. The apparent AUC was 0.849, with an estimated optimism of 0.026 and an optimism-corrected AUC of 0.823. The optimism-corrected Brier score, calibration intercept, and calibration slope were 0.169, 0.024, and 0.917, respectively. These results indicated only modest attenuation after optimism correction and supported the internal stability of the final model (**Supplementary Table 7**). Consistent results were observed in ROC analyses, where TabNet achieved the highest numerical AUC in the external validation cohort, in line with its favorable C-index performance (**Figures 3A, B**).

**Figure 3:**
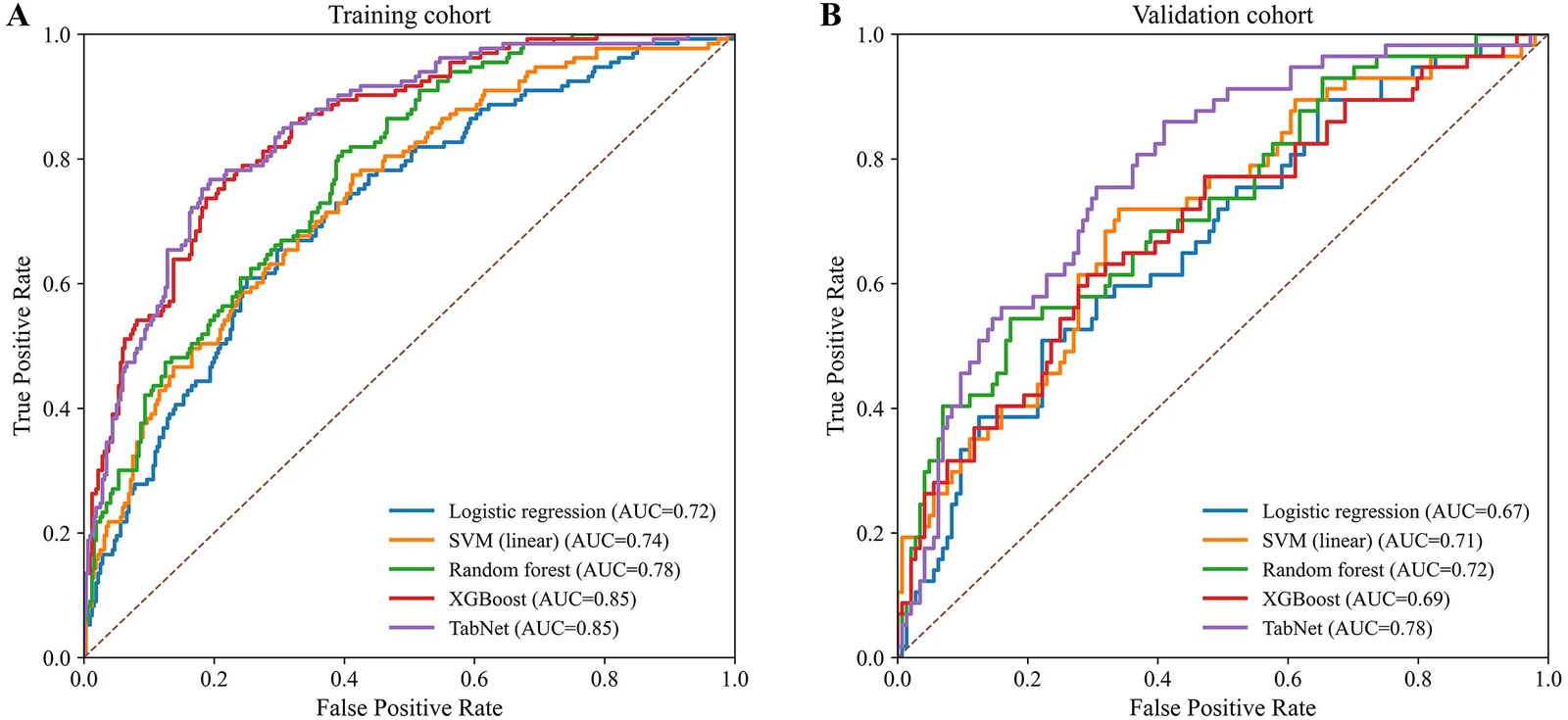
ROC curves of prediction models. **(A)** ROC curves of the five models in the training cohort; **(B)** ROC curves of the five models in the external validation cohort.

To further assess model robustness in the context of the limited number of outcome events relative to the original predictor set, a reduced-feature sensitivity analysis was performed using the 10 highest-ranked predictors identified by SHAP analysis within the training cohort. This reduced the number of predictors from 51 to 10 and increased the crude events-per-predictor ratio from 2.76 to 14.10. In the independent external validation cohort, the reduced-feature TabNet model retained acceptable discrimination, with an AUC of 0.75 (95% CI, 0.67–0.79), compared with 0.78 (95% CI, 0.71–0.85) for the full-feature model. The Brier score was 0.173, with a calibration intercept of 0.03 and a calibration slope of 0.88. These findings indicated only limited attenuation in discrimination with broadly preserved calibration after substantial predictor reduction, supporting the robustness of the principal predictive signal (**Supplementary Table 8**; **Supplementary Figures 1A, B**).

#### 3.2.3 Model calibration and clinical utility evaluation

The calibration performance of the final model was assessed in the external validation cohort. The calibration curve demonstrated an overall good agreement between predicted probabilities and observed event rates across most risk strata, particularly within the low- to intermediate-risk range (**Figure 4A**). To further evaluate the potential clinical utility of the model, DCA was performed. The model consistently yielded a higher net benefit than both the “treat-all” and “treat-none” strategies across a wide range of clinically relevant threshold probabilities, indicating favorable clinical applicability in decision-making (**Figure 4B**).

**Figure 4:**
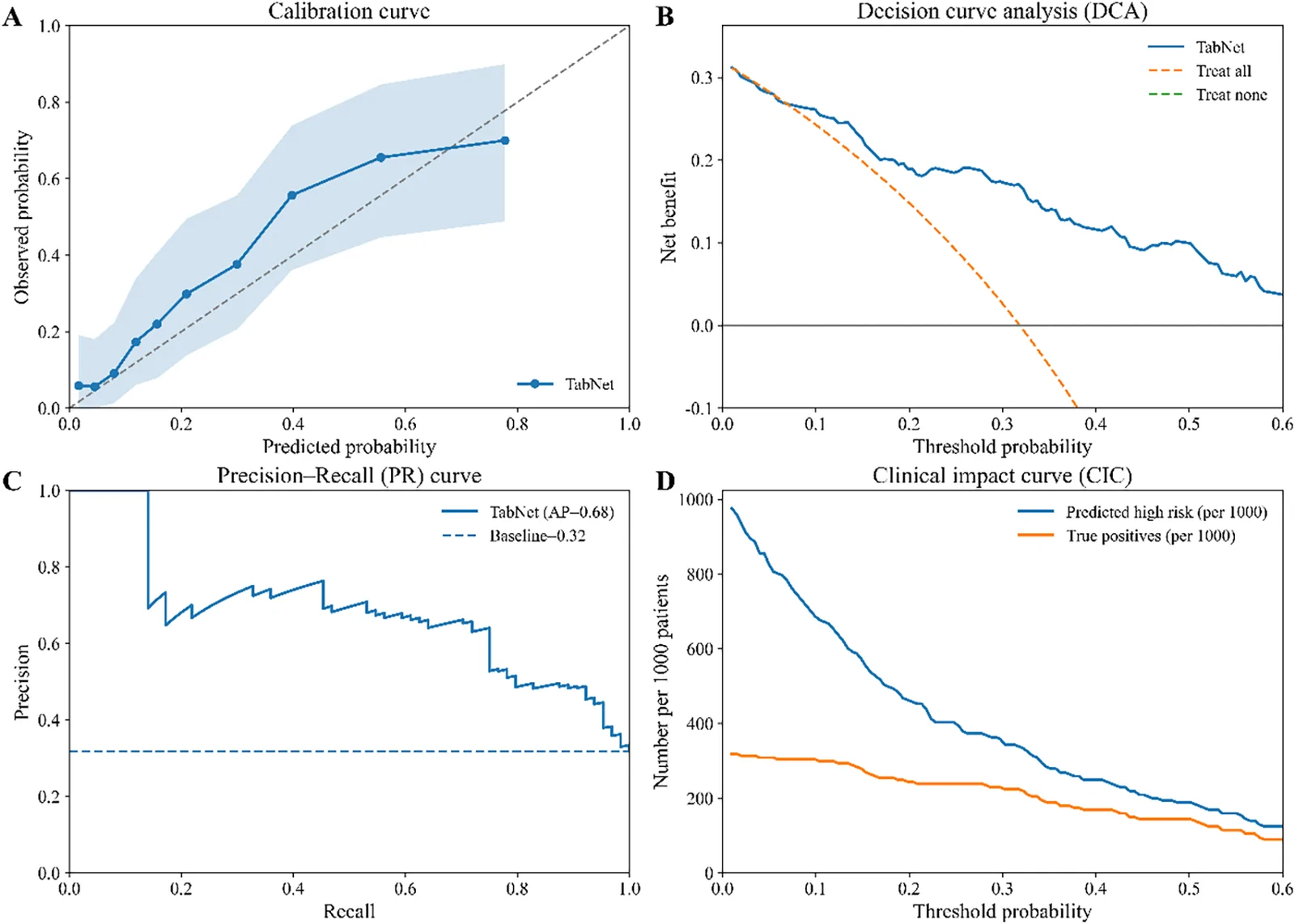
Model calibration and clinical utility assessment. **(A)** Calibration curve; **(B)** Decision curve analysis; **(C)** Precision–recall curve; **(D)** Clinical impact curve.

In addition, the PR curve showed that the model achieved a substantially higher average precision than the baseline event rate, suggesting improved identification of patients at elevated risk (**Figure 4C**). The CIC further illustrated that, across increasing threshold probabilities, the number of patients classified as high risk decreased gradually, while the number of true positive cases remained clinically meaningful, supporting the model’s potential value for risk stratification in practice (**Figure 4D**).

#### 3.2.4 Threshold-based risk stratification performance

To further evaluate the clinical interpretability of the final TabNet model, the continuous predicted probabilities were converted into binary threshold-based risk categories. Using the cut-off derived from the training cohort, patients in the external validation cohort were classified as high risk or non-high risk. In the external validation cohort, application of this fixed threshold classified 94 patients as high risk and 107 patients as non-high risk. Patients in the high-risk group had a markedly higher observed incidence of early clinically significant irAEs than those in the non-high-risk group (47/94, 50.0% vs. 12/107, 11.2%; P < 0.001; OR = 7.92, 95% CI: 3.84–16.35; **Figure 5**). At this threshold, the model achieved a sensitivity of 79.7%, specificity of 66.9%, positive predictive value of 50.0%, negative predictive value of 88.8%, accuracy of 70.6%, and F1 score of 61.4% in the external validation cohort. These findings suggest that the continuous model output can be translated into clinically interpretable risk categories with acceptable threshold-based classification performance.

**Figure 5:**
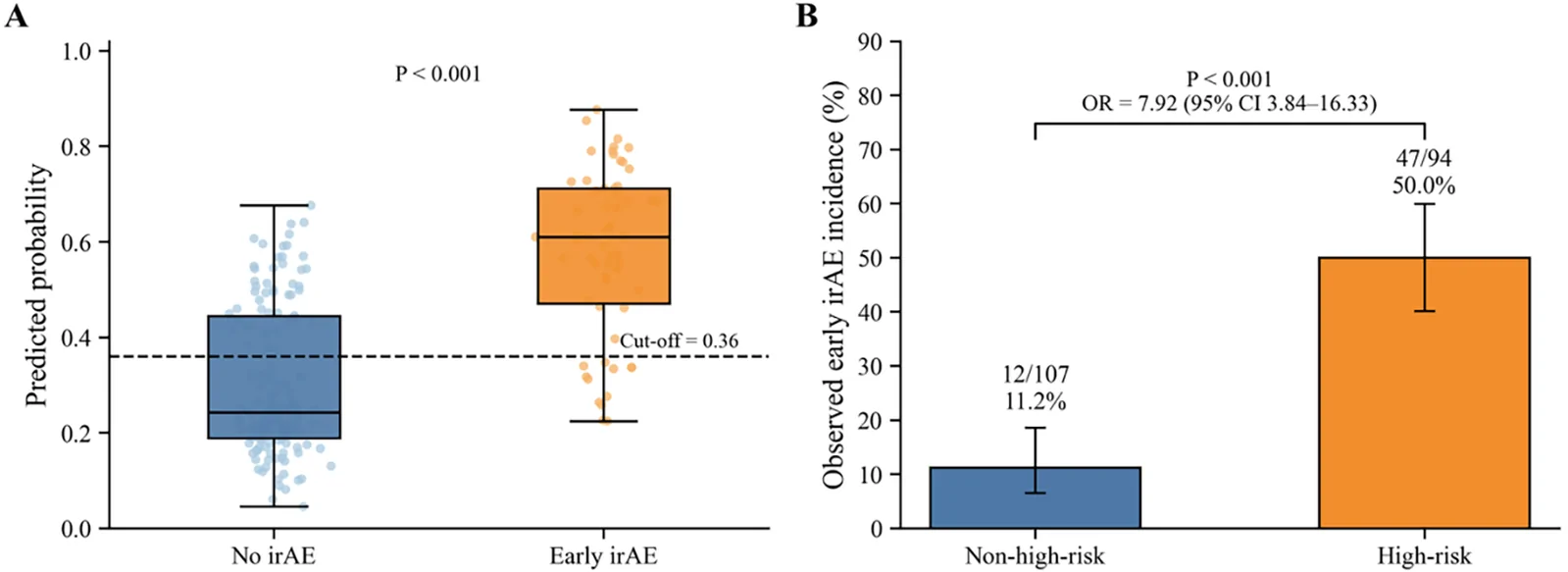
Threshold-based risk stratification performance. **(A)** Predicted probability distribution according to observed early irAE status; **(B)** Observed incidence of early irAEs in the threshold-defined high-risk and non-high-risk groups.

### 3.3 Model interpretability analysis

To first examine the intrinsic interpretability of the final TabNet model, attention-based feature masks were extracted and visualized. The global attention weights showed that the model assigned the highest average attention to CD4/CD8 ratio and IgG, followed by ALB, PLT, CD19-positive B-cell proportion, NLR, age, and BCLC stage (**Figure 6A**). The local feature-mask heatmap further demonstrated heterogeneous attention patterns across individual samples, suggesting that TabNet generated risk predictions by adaptively focusing on different combinations of baseline predictors at the patient level (**Figure 6B**).

**Figure 6:**
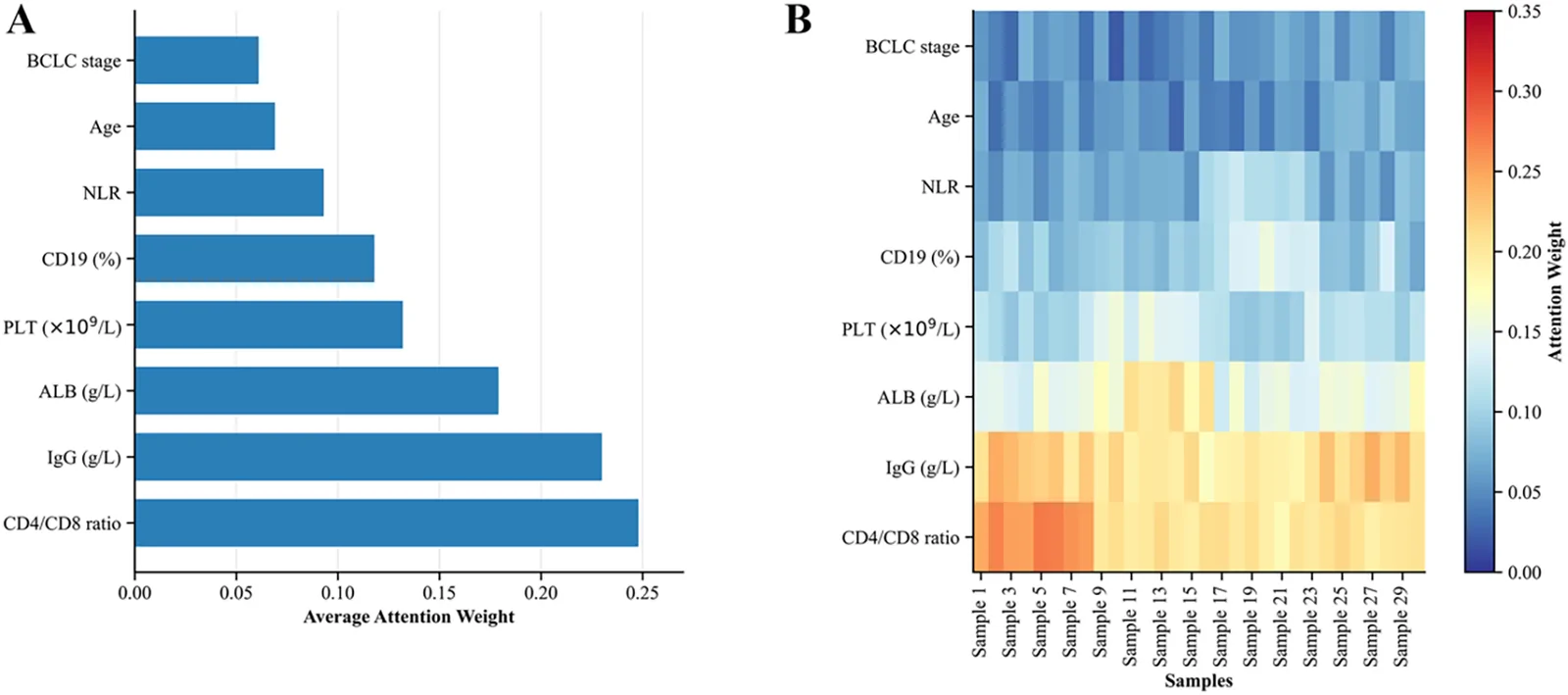
Attention-based feature-mask analysis of the final TabNet model. **(A)** Global feature importance based on average attention weights; **(B)** Local feature-mask heatmap showing sample-level attention weights for key baseline predictors.

To improve the interpretability of the final TabNet model, SHAP (Shapley Additive Explanations) analysis was conducted to quantify the contribution and effect direction of individual baseline variables on the prediction of early immune-related adverse events (irAE ≥ Grade 2). As shown in **Figure 7**, the SHAP summary plot provides a global overview of feature importance and inter-individual variability in model predictions. The CD4/CD8 ratio emerged as the most influential variable, followed by serum IgG levels, ALB, age, PLT, CD19-positive B cell proportion, and BCLC stage. Higher IgG levels, older age, increased CD19 (%), and more advanced BCLC stage were generally associated with positive SHAP values, indicating an increased predicted risk of early irAEs, whereas higher ALB levels and PLT counts tended to show negative SHAP values, indicating associations with lower predicted risk. Notably, the CD4/CD8 ratio displayed a wide distribution of SHAP values across both positive and negative ranges, reflecting substantial heterogeneity in its impact on individual predictions.

**Figure 7:**
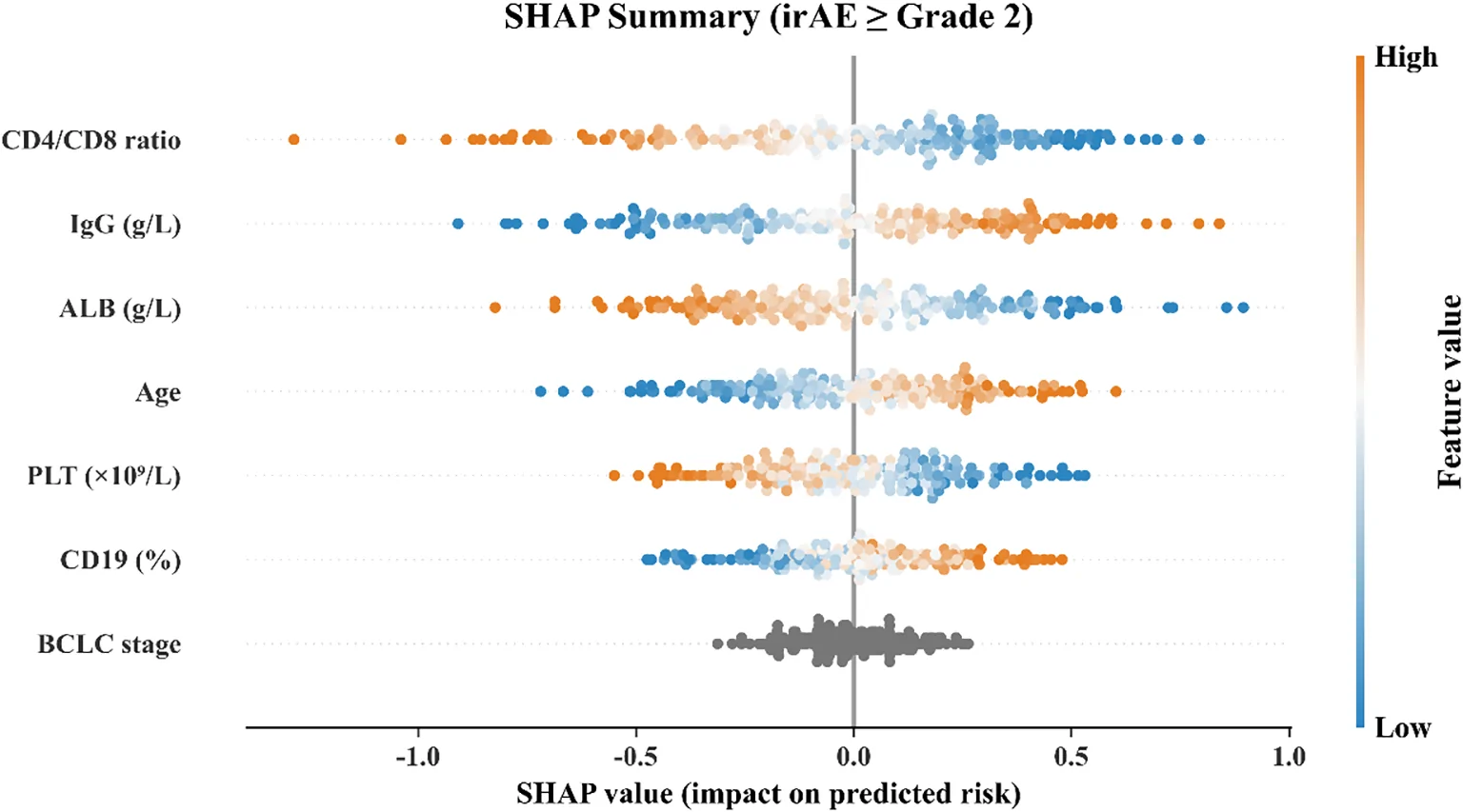
SHAP summary of baseline predictors for early irAE.

The CD4/CD8 ratio exhibited a clear inverse relationship with its SHAP values, indicating that increasing CD4/CD8 ratios were progressively associated with lower predicted risk of early irAEs (**Figure 8A**). This association was not uniform across individuals but appeared to vary according to IgG levels, suggesting that the predictive contribution of the CD4/CD8 ratio differed across IgG levels. In contrast, serum IgG demonstrated a predominantly positive and approximately linear association with SHAP values, with higher IgG concentrations corresponding to increased predicted risk, particularly among patients with lower CD4/CD8 ratios (**Figure 8B**). Together, these patterns suggest that cellular and humoral immune features may jointly contribute to the model’s risk stratification for early immune-related toxicity.

**Figure 8:**
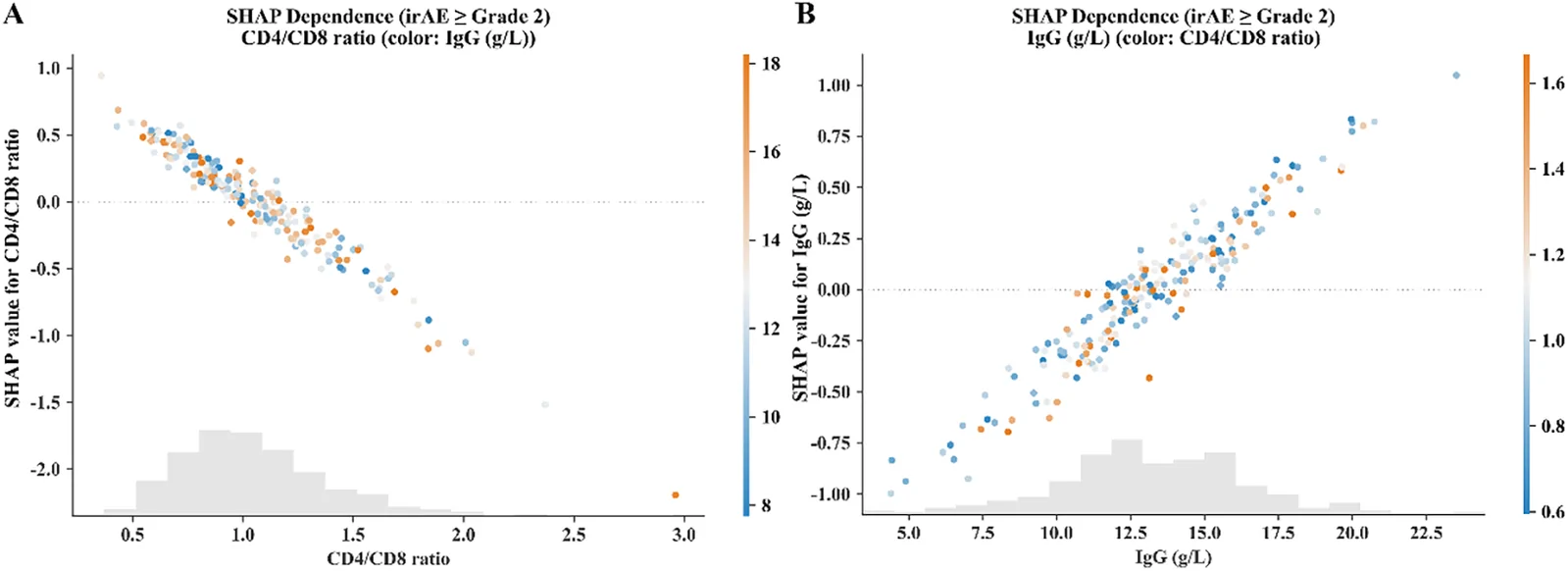
SHAP dependence plots of key immune-related predictors for early irAE. **(A)** Dependence of CD4/CD8 ratio colored by serum IgG levels; **(B)** Dependence of serum IgG levels colored by CD4/CD8 ratio.

### 3.4 Risk-stratified feature patterns and immune profiles

To further explore how baseline features jointly behaved across different risk strata defined by the final TabNet model, additional analyses were performed focusing on feature clustering within the high-risk group and SHAP-based risk stratification patterns. Hierarchical clustering of standardized baseline variables among patients classified as high risk revealed distinct co-variation patterns between clinical and immunological features (**Figure 9**). Immune-related parameters, including the CD4/CD8 ratio, serum IgG levels, and CD19-positive B cell proportion, tended to cluster together, whereas liver function–related markers such as albumin and platelet count formed a separate feature group. This structure suggests that, within the high-risk population, immune-related features and host functional reserve may represent partially independent dimensions associated with model-predicted early irAE risk.

**Figure 9:**
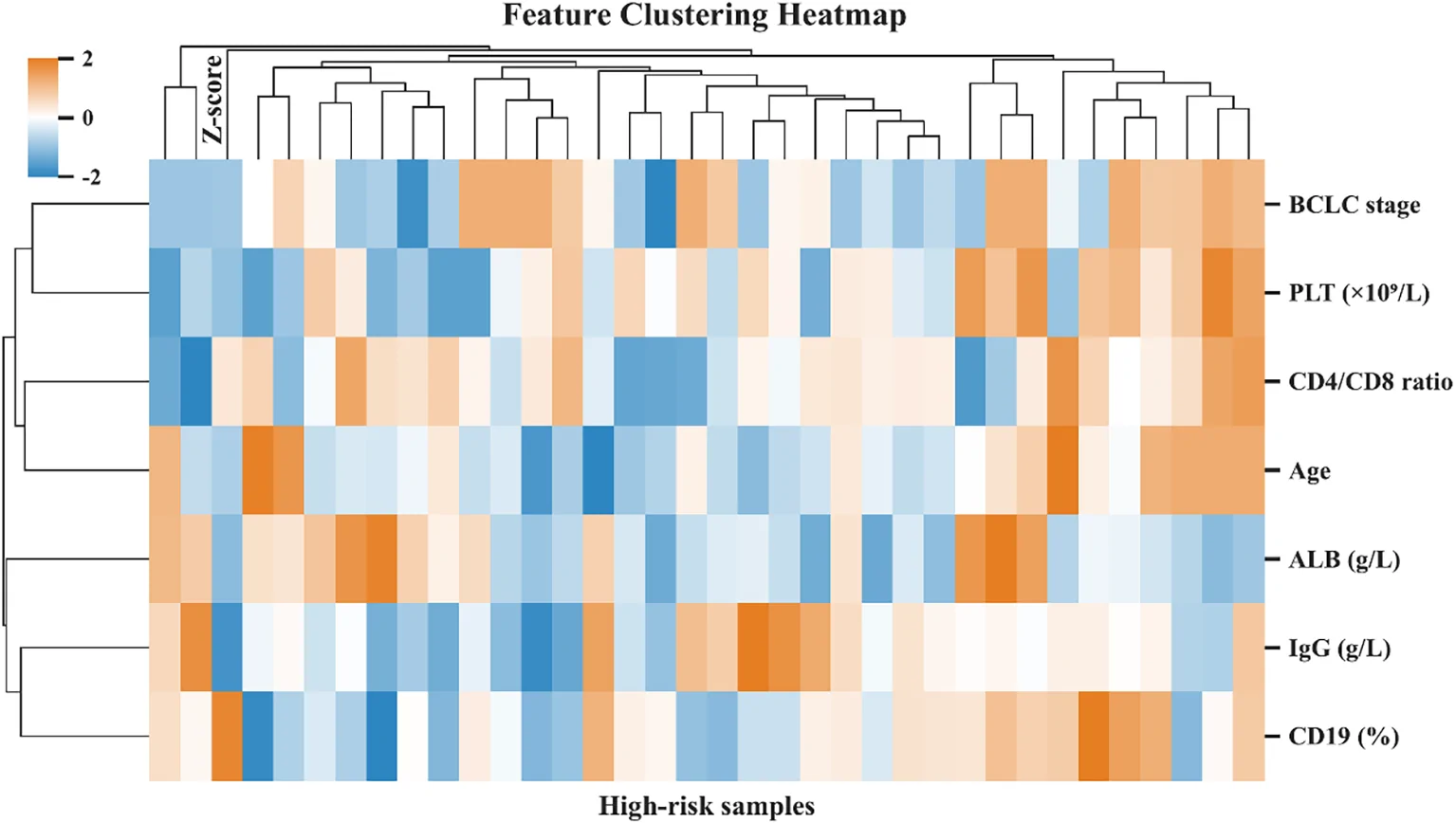
Feature clustering heatmap of baseline variables in the high-risk group.

Consistent with this observation, the risk-stratified SHAP profile demonstrated systematic differences in feature contributions across low-, intermediate-, and high-predicted-risk strata defined by predicted-probability tertiles (**Figure 10**). In the high-risk group, the CD4/CD8 ratio and IgG levels showed predominantly positive mean SHAP values, indicating a stronger contribution to increased predicted risk, whereas these effects were attenuated or reversed in the low-risk group. Albumin and platelet count exhibited progressively more negative SHAP values from high- to low-risk strata, indicating stronger associations with lower model-predicted risk. These graded shifts in SHAP contributions across risk strata indicate that the model captured not only individual feature contributions but also risk-dependent patterns of feature importance., These findings suggest that model-predicted early irAE risk was associated with coordinated patterns of immune status and host functional indicators, and that the TabNet model integrated these multidimensional patterns for risk stratification.

**Figure 10:**
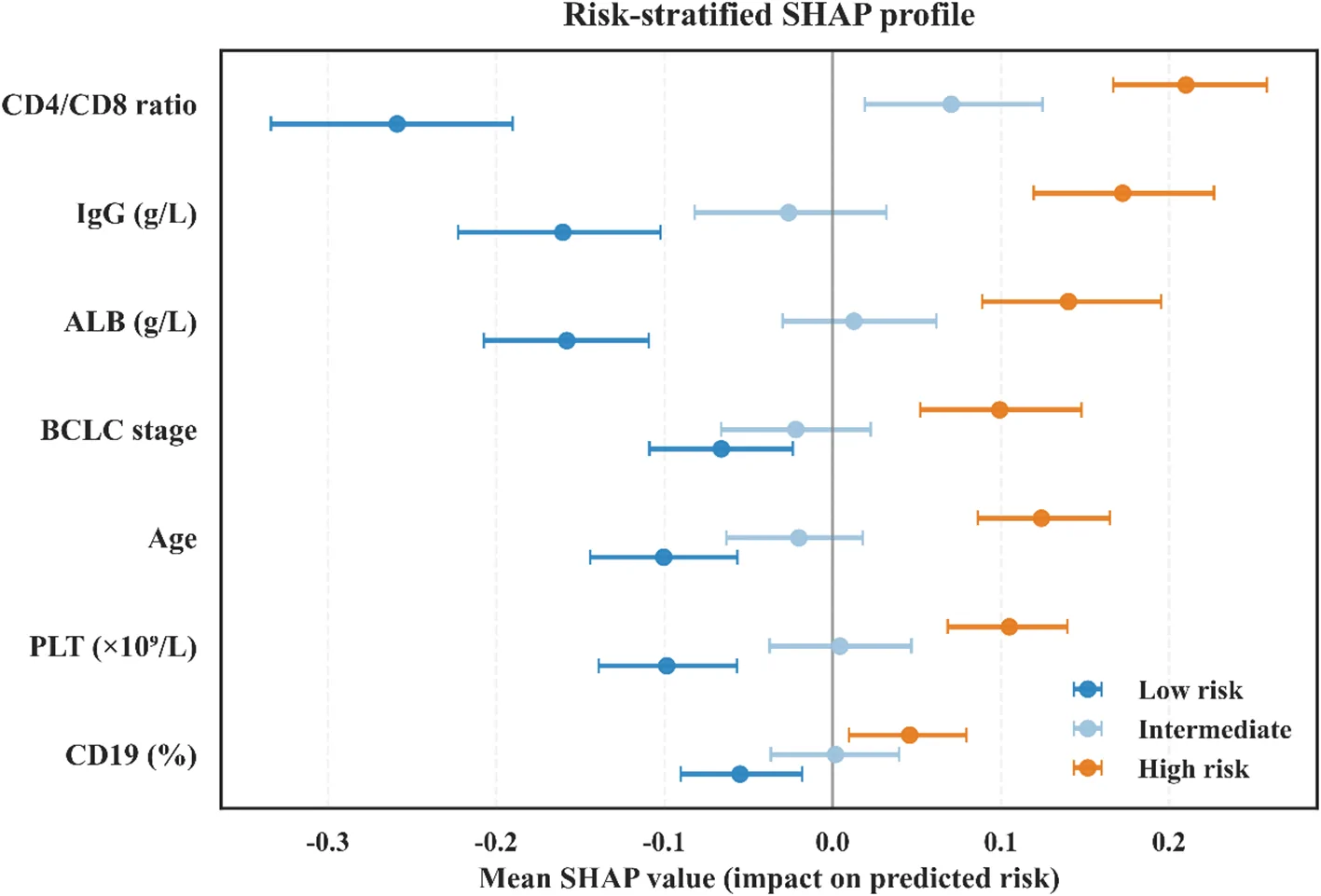
Risk-stratified SHAP profiles by predicted risk groups.

### 3.5 Subgroup analysis

To further evaluate the robustness of the final TabNet model across clinically relevant patient subsets, subgroup analyses were performed exclusively in the external validation cohort according to key baseline characteristics, including age, sex, Child–Pugh class, tumor size, BCLC stage, and surgical status (**Figure 11**). Model discrimination was assessed within each subgroup using AUC with corresponding 95% confidence intervals. Overall, the model demonstrated consistently acceptable discriminative performance across all predefined subgroups, with AUC values generally ranging from approximately 0.75 to 0.81. No marked deterioration in model performance was observed in any specific subgroup, suggesting generally consistent model performance across the external validation subgroups.

**Figure 11:**
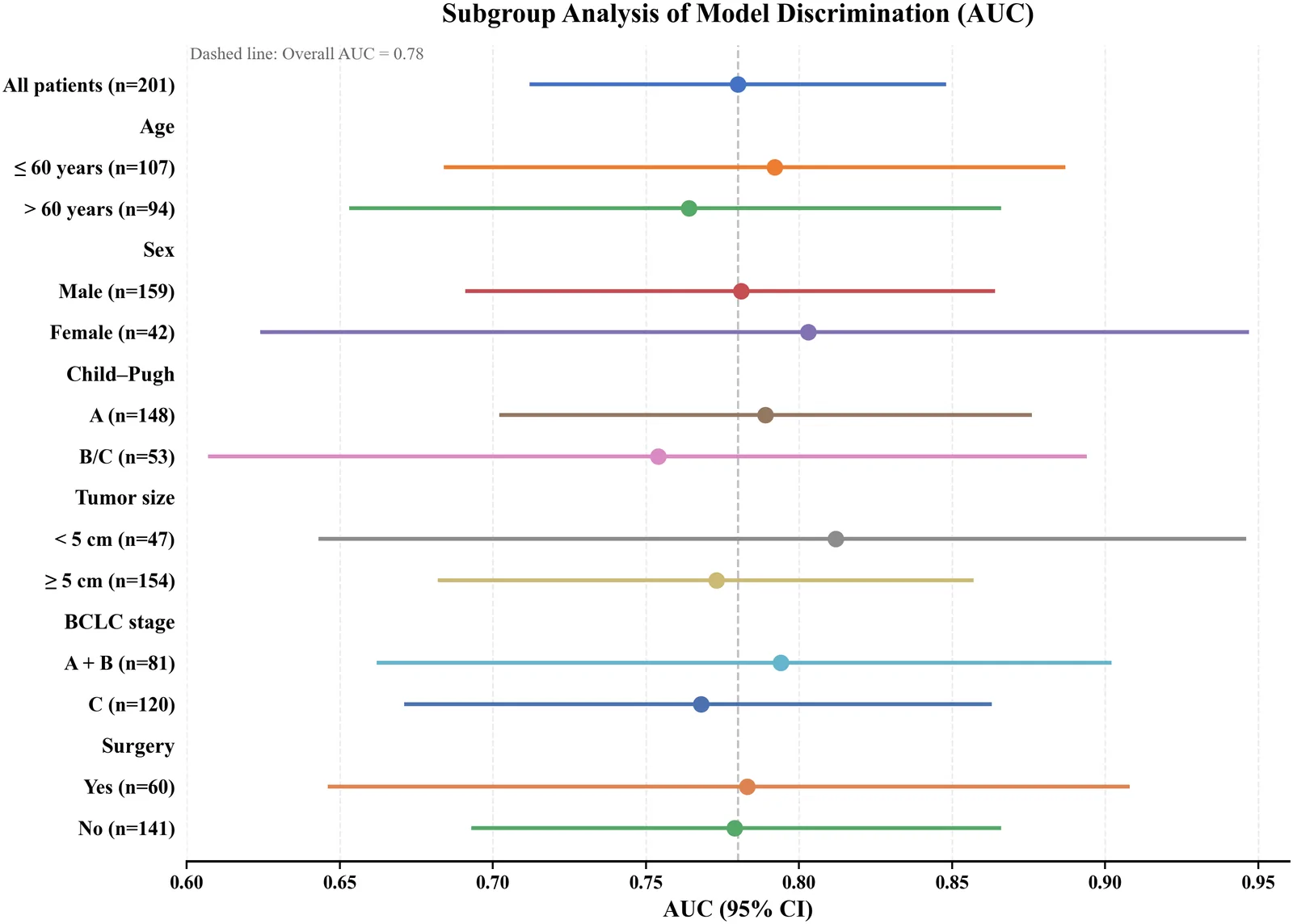
Subgroup analysis of model discrimination.

## 4 Discussion

In this multicenter retrospective study, we developed and externally validated an interpretable deep learning model to predict early-onset clinically significant immune-related adverse events (irAEs ≥ grade 2) in patients with hepatocellular carcinoma receiving ICIs-based therapy. By focusing on a pre-treatment and clinically actionable endpoint, this study extends beyond descriptive analyses of immune toxicity and establishes a structured framework for individualized risk stratification before immunotherapy initiation. The findings suggest that early irAE risk may be associated with coordinated baseline immune and host characteristics, supporting the feasibility of proactive risk assessment in real-world HCC populations.

Previous irAE risk-prediction studies have used inflammatory markers, clinical nomograms, and machine learning approaches to estimate irAE risk across different cancer populations. Inflammatory or hematological indicators, particularly the neutrophil-to-lymphocyte ratio, have been explored as accessible predictors of irAE susceptibility during immune checkpoint blockade (15, 16). Multivariable clinical models and nomograms have also been developed to estimate irAE risk by integrating clinical characteristics, treatment-related factors, and routine laboratory variables (17, 18). More recently, machine learning approaches have been applied to predict irAEs or organ-specific toxicities, including immune checkpoint inhibitor–related pneumonitis (19, 20). However, these studies remain heterogeneous in terms of tumor type, treatment regimen, toxicity definition, prediction time point, and validation strategy, and many were conducted in pan-cancer or non-HCC cohorts, focused on single biomarkers, or lacked independent external validation. Compared with these prior efforts, the present study focused on early clinically significant irAEs in patients with HCC receiving immune checkpoint inhibitor–based therapy, with atezolizumab plus bevacizumab serving as the representative regimen in this cohort. By integrating routinely available clinical variables with baseline circulating immunological markers and incorporating external validation, interpretable modeling, and threshold-based risk stratification, this study provides a disease-specific framework for pretreatment risk assessment in HCC immunotherapy.

We systematically compared five prediction models and found that TabNet achieved the most favorable overall balance between discrimination, calibration, and generalization, particularly in the independent external validation cohort. Although XGBoost demonstrated strong performance in the training cohort, the marked decline during external validation suggested potential overfitting. TabNet maintained relatively stable performance across cohorts despite a moderate sample size, suggesting favorable generalization. This stability may partly derive from its sequential attention mechanism, which adaptively selects informative features while enforcing sparsity constraints. Such architecture may reduce reliance on spurious correlations and enhance robustness in tabular clinical data with complex feature interactions.

In real-world studies of emerging treatment strategies, the relatively short duration of clinical use, restricted eligible populations, and requirements for complete baseline and follow-up data often limit the attainable sample size and number of outcome events. In the present study, 51 prespecified candidate predictors and 141 events yielded a crude events-per-candidate-predictor ratio of 2.76, indicating a potential risk of model instability and overfitting. The full prespecified predictor set was retained in the primary analysis because the candidate variables were defined *a priori* based on clinical relevance and biological plausibility, and variables with limited individual associations may still contribute to multivariable prediction through nonlinear relationships or interactions. Bootstrap internal validation of the final TabNet model showed only modest optimism, with the AUC decreasing from 0.849 to an optimism-corrected value of 0.823. The optimism-corrected Brier score, calibration intercept, and calibration slope were 0.169, 0.024, and 0.917, respectively. To further assess whether the predictive signal depended on the full predictor set, we performed a reduced-feature sensitivity analysis using the 10 highest-ranked SHAP predictors identified within the training cohort. This increased the crude events-per-predictor ratio from 2.76 to 14.10. Despite substantial predictor reduction, external discrimination showed only limited attenuation (AUC, 0.78 vs. 0.75), while the Brier score and calibration remained broadly preserved. These findings provide additional support that the principal predictive signal was retained within a more parsimonious feature set and was not dependent on the complete 51-variable input set. Nevertheless, the relatively limited number of outcome events remains an important constraint, and validation in larger prospective cohorts remains necessary.

Interpretability is essential for clinical adoption of machine learning models (21). The TabNet framework enabled multi-level interpretability through feature masks and SHAP analysis, allowing transparent assessment of both global feature importance and individual-level risk attribution (22–24). The alignment between attention-based feature selection and SHAP explanations facilitated consistent interpretation and supported individualized clinical reasoning rather than reliance on population averages (25).

The identified predictors provide biologically plausible context for the observed predictive associations with early immune-related toxicity. The CD4/CD8 ratio emerged as the most influential variable, highlighting its predictive relevance to early irAE risk and its potential relationship with baseline T-cell homeostasis (26, 27). Lower CD4/CD8 ratios may reflect relative cytotoxic T-cell predominance and heightened immune reactivity, providing a plausible biological context for their association with higher model-predicted irAE risk (28–30). Elevated serum IgG levels and increased CD19-positive B-cell proportions were also associated with higher model-predicted risk, consistent with previous observations linking humoral immune activity with immune-related inflammatory responses (31–33). In contrast, higher albumin levels and platelet counts were associated with lower model-predicted irAE risk and may serve as markers of preserved hepatic synthetic function and systemic reserve (34, 35). Collectively, these predictive patterns suggest that baseline immune status and host functional capacity provide complementary information for model-based early irAE risk stratification. From a temporal perspective, focusing specifically on early irAEs may improve the biological interpretability of pretreatment predictive associations. Previous studies have suggested that toxicities occurring during the initial treatment cycles may be more closely related to pre-existing immune susceptibility, whereas later irAEs may increasingly reflect cumulative immune changes associated with prolonged treatment, including sustained immune stimulation, epitope spreading, or progressive disruption of peripheral tolerance (36, 37). Modeling early events separately therefore provides a prediction framework more closely aligned with pretreatment host characteristics while reducing the potential influence of later treatment-related immune changes. Importantly, attention weights and SHAP values quantify the contribution of individual variables to model predictions and should not be interpreted as estimates of causal effects. Accordingly, the identified variables should be regarded as predictive correlates and hypothesis-generating biomarkers rather than established causal determinants of irAE development.

The risk-stratified clustering analysis revealed structured separation between immune-related parameters and markers of liver function and systemic reserve. This pattern suggests that baseline immune status and host functional reserve may represent two complementary dimensions associated with early irAE risk stratification. Based on these findings, we propose a dual-axis conceptual framework to interpret the heterogeneity of model-predicted early irAE risk. In this framework, one axis reflects baseline immune activation potential, captured by T-cell balance and humoral immune markers, whereas the second axis reflects host functional reserve, represented by hepatic synthetic function and systemic resilience. Patients with different combinations of immune activation and host reserve may therefore present distinct predicted-risk patterns for early clinically significant irAEs. This framework provides a biologically plausible and hypothesis-generating perspective for interpreting prediction-relevant heterogeneity in early irAE risk observed in clinical practice (38–40).

From a clinical perspective, the model offers a practical tool for pre-treatment risk stratification using routinely available laboratory and immunological parameters. Identification of high-risk individuals may facilitate intensified monitoring during the early treatment phase, including closer assessment of liver function, thyroid function, blood counts, inflammatory markers, and symptoms suggestive of immune-related toxicity. High-risk classification may also support timely multidisciplinary communication and informed patient counseling before therapy initiation. Importantly, this risk stratification framework is intended to support proactive monitoring and early recognition of irAEs rather than to compromise therapeutic intent or determine treatment exclusion. The model also highlights the value of baseline circulating immune profiling in anticipating immune-related toxicity and supports shared decision-making.

Several limitations should be acknowledged. First, although external validation was performed, the retrospective design and complete-case analysis may introduce residual selection bias and limit causal inference. The exclusion of patients with incomplete follow-up may also have introduced potential informative censoring and associated selection bias. Residual confounding from recent locoregional therapies or changes in antiviral treatment before systemic therapy could not be completely excluded, as these interventions may transiently affect liver function, inflammatory markers, and circulating immune parameters. Second, the sample size, while comparable to many real-world immunotherapy studies in HCC, may limit the detection of rare irAE subtypes and precludes organ-specific toxicity modeling. In addition, the limited number of events relative to the number of candidate predictors may increase the risk of model instability and overfitting, despite the relatively modest optimism observed in bootstrap internal validation and the stable performance of TabNet in the external validation cohort. Adjudication of irAEs in patients with severely impaired liver function may also be challenging because hepatic decompensation, disease progression, and immune-related liver injury can present with overlapping clinical and laboratory features. Third, all patients in the present cohort received atezolizumab plus bevacizumab; therefore, the generalizability of the model to other HCC immunotherapy regimens requires further validation. Fourth, the immunological variables were derived from routine peripheral blood measurements and may not fully capture intratumoral immune dynamics; residual inter-center variability in immunological testing procedures also cannot be completely excluded. Finally, although the model provides individualized predicted probabilities and clinically interpretable risk categories, its direct bedside use will require further prospective validation and practical implementation tools, such as a web-based calculator, mobile application, or electronic medical record–integrated decision support system. Mechanistic validation through experimental or longitudinal studies is also needed to confirm the biological pathways suggested by the predictive model.

## 5 Conclusion

This study developed and externally validated an interpretable deep learning model for predicting early-onset clinically significant immune-related adverse events in patients with hepatocellular carcinoma receiving ICIs-based therapy. By integrating routinely available clinical variables with baseline circulating immune parameters, the model demonstrated favorable predictive performance, stable external validation performance, and meaningful clinical interpretability.

## Data Availability

The raw data supporting the conclusions of this article will be made available by the authors, without undue reservation.
