# Supplementary material for: Development and external validation of an interpretable deep learning model for early immune-related adverse events in hepatocellular carcinoma
Source: Front Immunol. 2026 Sep 21;17:1886946. doi: 10.3389/fimmu.2026.1886946 (PMC13635132; doi:10.3389/fimmu.2026.1886946)
Supplement: Supplementary file 1 [file SupplementaryFile1.docx]

**Supplementary Figure 1** Sensitivity analysis of the reduced-feature TabNet model

(A) ROC curve of the reduced-feature TabNet model in the external validation cohort; (B) calibration curve of the reduced-feature TabNet model in the external validation cohort.

**Supplementary Table 1** Hyperparameter tuning and final selected parameters of the prediction models

| **Model** | **Class imbalance handling** | **Early stopping** | **Key tuning range** | **Final selected parameters** |
| --- | --- | --- | --- | --- |
| TabNet | Class-weighted loss | Yes; patience = 30 epochs | n_d/n_a: 8–32; n_steps: 3–5; gamma: 1.0–1.8; lambda_sparse: 1e-5–1e-3; learning rate: 0.005–0.02 | n_d = 16; n_a = 16; n_steps = 4; gamma = 1.5; lambda_sparse = 1e-4; learning rate = 0.02 |
| Logistic regression | class_weight = balanced | Not applicable | penalty: L1/L2; C: 0.01–100 | penalty = L2; C = 0.1 |
| Random forest | class_weight = balanced | Not applicable | n_estimators: 200–1000; max_depth: 3–7; min_samples_leaf: 1–10; max_features: sqrt/log2 | n_estimators = 500; max_depth = 5; min_samples_leaf = 5; max_features = sqrt |
| XGBoost | scale_pos_weight = 2.21 | Yes; early stopping rounds = 30 | n_estimators: 100–500; learning rate: 0.01–0.1; max_depth: 2–5; subsample: 0.7–1.0; colsample_bytree: 0.7–1.0; reg_alpha: 0–1; reg_lambda: 1–10 | n_estimators = 500; learning rate = 0.03; max_depth = 3; subsample = 0.8; colsample_bytree = 0.8; reg_alpha = 0.1; reg_lambda = 5; scale_pos_weight = 2.21 |
| Support vector machine | class_weight = balanced | Not applicable | kernel: linear/RBF; C: 0.1–100; gamma: scale/0.01–1 | kernel = radial basis function; C = 1; gamma = scale |

**Note:** Hyperparameter tuning was performed exclusively within the training cohort using stratified 5-fold cross-validation, with AUC as the tuning metric. The external validation cohort was not used for hyperparameter tuning or model selection. The random seed was fixed at 42 during model development, and the optimized models were then applied to the external validation cohort without further adjustment.

**Supplementary Table 2** Clinical profiles of early clinically significant irAEs

| **irAE category** | **Training cohort n (%)** | **External validation cohort n (%)** | **Grade 2 n** | **Grade 3 n** | **Grade 4 n** | **Median time to onset, days** | **Glucocorticoid use n (%)** | **Treatment interruption n (%)** | **Permanent discontinuation n (%)** |
| --- | --- | --- | --- | --- | --- | --- | --- | --- | --- |
| Hepatic toxicity | 50 (35.5) | 18 (30.5) | 50 | 16 | 2 | 45 | 34 (50.0) | 22 (32.4) | 6 (8.8) |
| Cutaneous toxicity | 27 (19.1) | 14 (23.7) | 35 | 6 | 0 | 28 | 12 (29.3) | 6 (14.6) | 0 (0.0) |
| Endocrine toxicity | 25 (17.7) | 9 (15.3) | 32 | 2 | 0 | 56 | 3 (8.8) | 2 (5.9) | 0 (0.0) |
| Pneumonitis | 9 (6.4) | 6 (10.2) | 9 | 5 | 1 | 62 | 12 (80.0) | 8 (53.3) | 3 (20.0) |
| Gastrointestinal toxicity / colitis | 9 (6.4) | 3 (5.1) | 9 | 3 | 0 | 36 | 8 (66.7) | 5 (41.7) | 1 (8.3) |
| Renal toxicity | 4 (2.8) | 3 (5.1) | 6 | 1 | 0 | 49 | 4 (57.1) | 2 (28.6) | 0 (0.0) |
| Hematologic toxicity | 7 (5.0) | 3 (5.1) | 6 | 3 | 1 | 24 | 4 (40.0) | 4 (40.0) | 1 (10.0) |
| Other irAEs | 10 (7.1) | 3 (5.1) | 11 | 2 | 0 | 40 | 5 (38.5) | 4 (30.8) | 1 (7.7) |
| Total | 141 (100.0) | 59 (100.0) | 158 | 38 | 4 | 42 | 82 (41.0) | 53 (26.5) | 12 (6.0) |

**Note:** Percentages in the training and external validation cohorts were calculated using the number of patients with early clinically significant irAEs in each cohort as the denominator. Percentages for glucocorticoid use, treatment interruption, and permanent discontinuation were calculated using the number of events in each irAE category as the denominator. For patients with multiple early irAEs, CTCAE grade and management information were summarized according to the highest-grade or clinically dominant early irAE.

**Supplementary Table 3** Baseline characteristics of candidate variables for model development

| **Items** | **Training cohort (n = 453)** | **External validation cohort (n = 201)** | ***P*** |
| --- | --- | --- | --- |
| Age (years), mean (SD) | 57.44 (10.38) | 56.20 (12.00) | 0.205 |
| BMI (Kg/m^2^), mean (SD) | 23.97 (3.94) | 24.40 (4.80) | 0.286 |
| Sex |  |  | 0.363 |
| Male | 372 (82.1%) | 159 (79.1%) |  |
| Female | 81 (17.9%) | 42 (20.9%) |  |
| Smoking |  |  | 0.129 |
| Yes | 93 (20.5%) | 52 (25.9%) |  |
| No | 360 (79.5%) | 149 (74.1%) |  |
| Drinking |  |  | 0.901 |
| Yes | 57 (12.6%) | 26 (12.9%) |  |
| No | 396 (87.4%) | 175 (87.1%) |  |
| Child pugh class |  |  | 0.865 |
| A | 341 (75.3%) | 148 (73.6%) |  |
| B | 95 (21.0%) | 44 (21.9%) |  |
| C | 17 (3.7%) | 9 (4.5%) |  |
| ABO blood type |  |  | 0.686 |
| A | 126 (27.8%) | 49 (24.4%) |  |
| B | 135 (29.8%) | 68 (33.8%) |  |
| AB | 66 (14.6%) | 27 (13.4%) |  |
| O | 126 (27.8%) | 57 (28.4%) |  |
| Surgery |  |  | 0.323 |
| Yes | 153 (33.8%) | 60 (29.9%) |  |
| No | 300 (66.2%) | 141 (70.1%) |  |
| Tumor number |  |  | 0.950 |
| Single | 186 (41.1%) | 82 (40.8%) |  |
| Multiple | 267 (58.9%) | 119 (59.2%) |  |
| Tumor size |  |  | 0.154 |
| < 5 cm | 84 (18.5%) | 47 (23.4%) |  |
| ≥ 5 cm | 369 (81.5%) | 154 (76.6%) |  |
| Liver cirrhosis |  |  | 0.367 |
| Yes | 135 (29.8%) | 67 (33.3%) |  |
| No | 318 (70.2%) | 134 (66.7%) |  |
| BCLC stage |  |  | 0.416 |
| A + B | 198 (43.7%) | 81 (40.3%) |  |
| C | 255 (56.3%) | 120 (59.7%) |  |
| Early irAE (grade ≥2) |  |  | 0.650 |
| Yes | 141 (31.1%) | 59 (29.4%) |  |
| No | 312 (68.9%) | 142 (70.6%) |  |
| IgA (g/L, mean [SD]) | 2.38 (0.82) | 2.42 (0.95) | 0.611 |
| IgG (g/L, mean [SD]) | 13.10 (3.05) | 12.75 (3.72) | 0.249 |
| IgM (g/L, mean [SD]) | 1.18 (0.49) | 1.21 (0.58) | 0.497 |
| CD3 (+) (%) | 80.6 (8.6) | 79.8 (9.9) | 0.214 |
| CD4 (+) (%) | 40.9 (8.8) | 39.6 (9.4) | 0.356 |
| CD8 (+) (%) | 25.3 (9.7) | 26.1 (10.3) | 0.335 |
| CD4 (+) / CD8 (+) | 2.00 (1.09) | 1.88 (1.12) | 0.191 |
| CD3 (+) CD4 (+) CD8 (+) (%) | 0.52 (0.36) | 0.58 (1.29) | 0.546 |
| CD19 (+) (%) | 9.6 (3.3) | 10.2 (4.1) | 0.084 |
| CD3 (-) CD16 (+) CD56 (+) (%) | 18.7 (9.8) | 19.4 (11.2) | 0.455 |
| CD3 (+) CD16 (+) CD56 (+) (%) | 3.3 (3.4) | 2.8 (3.7) | 0.111 |
| ALT (U/L, median [IQR]) | 14 (9, 25) | 16 (11, 29) | 0.233 |
| AST (U/L, median [IQR]) | 20 (15, 26) | 22 (16, 31) | 0.198 |
| γ-GGT (U/L, median [IQR]) | 23 (15, 44) | 27 (17, 52) | 0.089 |
| LDH (U/L, median [IQR]) | 171 (145, 212) | 178 (150, 226) | 0.312 |
| TBIL (μmol/L, median [IQR]) | 12.0 (9.0, 15.2) | 13.1 (9.8, 17.4) | 0.141 |
| DBIL (μmol/L, median [IQR]) | 2.7 (1.9, 3.5) | 2.9 (2.0, 3.9) | 0.227 |
| IDBIL (μmol/L, median [IQR]) | 9.0 (6.9, 11.8) | 9.8 (7.4, 13.6) | 0.168 |
| TP (g/L, mean [SD]) | 69.9 (7.1) | 70.6 (7.9) | 0.274 |
| ALB (g/L, mean [SD]) | 39.0 (4.4) | 38.5 (4.9) | 0.215 |
| GLOB (g/L, mean [SD]) | 30.1 (5.1) | 31.0 (5.9) | 0.062 |
| A/G, mean (SD) | 1.33 (0.25) | 1.30 (0.27) | 0.181 |
| PALB (mg/L, mean [SD]) | 199.6 (65.3) | 210.8 (72.4) | 0.061 |
| BUN (mmol/L, mean [SD]) | 5.52 (1.60) | 5.75 (1.92) | 0.139 |
| CREA (μmol/L, mean [SD]) | 76.1 (16.6) | 78.0 (18.9) | 0.219 |
| UA (μmol/L, mean [SD]) | 294.7 (83.7) | 305.6 (94.1) | 0.054 |
| ALP (U/L, median [IQR]) | 90 (72, 118) | 97 (75, 131) | 0.164 |
| Glu (mmol/L, median [IQR]) | 5.10 (4.6, 5.8) | 5.15 (4.7, 5.9) | 0.742 |
| WBC (10^9^/L, median [IQR]) | 6.4 (5.0, 8.1) | 6.9 (5.4, 8.9) | 0.121 |
| NEU (10^9^/L, median [IQR]) | 3.9 (2.8, 5.4) | 4.3 (3.0, 6.2) | 0.093 |
| LYM (10^9^/L, median [IQR]) | 1.65 (1.24, 1.99) | 1.58 (1.18, 2.02) | 0.417 |
| MON (10^9^/L, median [IQR]) | 0.48 (0.32, 0.62) | 0.53 (0.35, 0.70) | 0.156 |
| RBC (10^12^/L, mean [SD]) | 4.27 (0.62) | 4.22 (0.66) | 0.363 |
| HGB (g/L, mean [SD]) | 122.2 (21.8) | 118.6 (24.1) | 0.071 |
| HCT (L/L, mean [SD]) | 37.9 (6.4) | 36.8 (7.2) | 0.063 |
| PLT (10^9^/L, median [IQR]) | 236 (184, 312) | 255 (190, 338) | 0.094 |
| INR, mean (SD) | 1.00 (0.13) | 1.01 (0.15) | 0.414 |
| Fbg (g/L, median [IQR]) | 3.47 (2.80, 4.36) | 3.62 (2.95, 4.78) | 0.163 |
| Ddi (ng/L, median [IQR]) | 0.75 (0.41, 1.58) | 0.88 (0.48, 1.92) | 0.087 |

**Table note:** BMI, body mass index; irAE, immune-related adverse event; IgA, immunoglobulin A; IgG, immunoglobulin G; IgM, immunoglobulin M; CD, cluster of differentiation; ALT, alanine aminotransferase; AST, aspartate aminotransferase; γ-GGT, gamma-glutamyl transferase; LDH, lactate dehydrogenase; TBIL, total bilirubin; DBIL, direct bilirubin; IDBIL, indirect bilirubin; TP, total protein; ALB, albumin; GLOB, globulin; A/G, albumin-to-globulin ratio; PALB, prealbumin; BUN, blood urea nitrogen; CREA, creatinine; UA, uric acid; ALP, alkaline phosphatase; Glu, glucose; WBC, white blood cell count; NEU, neutrophil count; LYM, lymphocyte count; MON, monocyte count; RBC, red blood cell count; HGB, hemoglobin; HCT, hematocrit; PLT, platelet count; INR, international normalized ratio; Fbg, fibrinogen; D-dimer, fibrin degradation product D-dimer; BCLC, Barcelona Clinic Liver Cancer; IQR, interquartile range; SD, standard deviation.

**Supplementary Table 4** Comparison of baseline characteristics between the two external validation centers

| **Items** | **Chongqing center (n = 170)** | **Harbin center (n = 31)** | ***P*** | **Absolute SMD** |
| --- | --- | --- | --- | --- |
| Age (years), mean (SD) | 55.94 (11.80) | 57.60 (13.00) | 0.511 | 0.138 |
| BMI (kg/m²), mean (SD) | 24.49 (4.70) | 23.90 (5.20) | 0.559 | 0.123 |
| Sex |  |  | 0.465 | 0.139 |
| Male | 136 (80.0%) | 23 (74.2%) |  |  |
| Female | 34 (20.0%) | 8 (25.8%) |  |  |
| Smoking |  |  | 0.368 | 0.183 |
| Yes | 46 (27.1%) | 6 (19.4%) |  |  |
| No | 124 (72.9%) | 25 (80.6%) |  |  |
| Drinking |  |  | 0.773 | 0.120 |
| Yes | 23 (13.5%) | 3 (9.7%) |  |  |
| No | 147 (86.5%) | 28 (90.3%) |  |  |
| Child–Pugh class |  |  | 0.834 | 0.110 |
| A | 126 (74.1%) | 22 (71.0%) |  |  |
| B | 37 (21.8%) | 7 (22.6%) |  |  |
| C | 7 (4.1%) | 2 (6.5%) |  |  |
| ABO blood type |  |  | 0.995 | 0.052 |
| A | 41 (24.1%) | 8 (25.8%) |  |  |
| B | 58 (34.1%) | 10 (32.3%) |  |  |
| AB | 23 (13.5%) | 4 (12.9%) |  |  |
| O | 48 (28.2%) | 9 (29.0%) |  |  |
| Surgery |  |  | 0.750 | 0.062 |
| Yes | 50 (29.4%) | 10 (32.3%) |  |  |
| No | 120 (70.6%) | 21 (67.7%) |  |  |
| Tumor number |  |  | 0.888 | 0.027 |
| Single | 69 (40.6%) | 13 (41.9%) |  |  |
| Multiple | 101 (59.4%) | 18 (58.1%) |  |  |
| Tumor size |  |  | 0.419 | 0.153 |
| <5 cm | 38 (22.4%) | 9 (29.0%) |  |  |
| ≥5 cm | 132 (77.6%) | 22 (71.0%) |  |  |
| Liver cirrhosis |  |  | 0.49 | 0.133 |
| Yes | 55 (32.4%) | 12 (38.7%) |  |  |
| No | 115 (67.6%) | 19 (61.3%) |  |  |
| BCLC stage |  |  | 0.548 | 0.117 |
| A + B | 67 (39.4%) | 14 (45.2%) |  |  |
| C | 103 (60.6%) | 17 (54.8%) |  |  |
| IgA (g/L), mean (SD) | 2.41 (0.93) | 2.48 (1.04) | 0.728 | 0.074 |
| IgG (g/L), mean (SD) | 12.68 (3.65) | 13.15 (4.06) | 0.551 | 0.127 |
| IgM (g/L), mean (SD) | 1.20 (0.56) | 1.25 (0.66) | 0.694 | 0.087 |
| CD3+ (%), mean (SD) | 79.96 (9.70) | 78.90 (10.80) | 0.613 | 0.107 |
| CD4+ (%), mean (SD) | 39.44 (9.30) | 40.50 (10.00) | 0.586 | 0.113 |
| CD8+ (%), mean (SD) | 26.23 (10.20) | 25.40 (10.90) | 0.696 | 0.081 |
| CD4+/CD8+, mean (SD) | 1.86 (1.10) | 1.98 (1.20) | 0.607 | 0.108 |
| CD3+CD4+CD8+ (%), mean (SD) | 0.57 (1.20) | 0.64 (1.70) | 0.828 | 0.054 |
| CD19+ (%), mean (SD) | 10.29 (4.00) | 9.70 (4.60) | 0.507 | 0.144 |
| CD3−CD16+CD56+ (%), mean (SD) | 19.22 (11.00) | 20.40 (12.20) | 0.618 | 0.105 |
| CD3+CD16+CD56+ (%), mean (SD) | 2.75 (3.60) | 3.10 (4.10) | 0.659 | 0.095 |
| ALT (U/L), median (IQR) | 16 (11, 28) | 18 (12, 31) | 0.465 | 0.156 |
| AST (U/L), median (IQR) | 22 (16, 30) | 24 (17, 34) | 0.410 | 0.186 |
| γ-GGT (U/L), median (IQR) | 26 (17, 50) | 31 (18, 57) | 0.371 | 0.199 |
| LDH (U/L), median (IQR) | 176 (149, 223) | 188 (154, 241) | 0.336 | 0.213 |
| TBIL (μmol/L), median (IQR) | 12.9 (9.7, 17.0) | 14.0 (10.3, 18.8) | 0.367 | 0.198 |
| DBIL (μmol/L), median (IQR) | 2.8 (2.0, 3.8) | 3.1 (2.2, 4.2) | 0.299 | 0.221 |
| IDBIL (μmol/L), median (IQR) | 9.6 (7.3, 13.4) | 10.5 (7.8, 14.5) | 0.353 | 0.196 |
| TP (g/L), mean (SD) | 70.47 (7.80) | 71.30 (8.50) | 0.616 | 0.105 |
| ALB (g/L), mean (SD) | 38.37 (4.80) | 39.20 (5.20) | 0.413 | 0.171 |
| GLOB (g/L), mean (SD) | 31.07 (5.80) | 30.60 (6.40) | 0.705 | 0.08 |
| A/G, mean (SD) | 1.29 (0.26) | 1.33 (0.29) | 0.477 | 0.151 |
| PALB (mg/L), mean (SD) | 209.3 (70.5) | 219.0 (81.0) | 0.536 | 0.134 |
| BUN (mmol/L), mean (SD) | 5.71 (1.85) | 5.98 (2.30) | 0.540 | 0.140 |
| CREA (μmol/L), mean (SD) | 77.6 (18.5) | 80.2 (21.0) | 0.523 | 0.138 |
| UA (μmol/L), mean (SD) | 304.1 (92.0) | 314.0 (105.0) | 0.626 | 0.105 |
| ALP (U/L), median (IQR) | 95 (74, 128) | 106 (79, 143) | 0.232 | 0.267 |
| Glu (mmol/L), median (IQR) | 5.10 (4.7, 5.9) | 5.30 (4.8, 6.0) | 0.256 | 0.225 |
| WBC (10⁹/L), median (IQR) | 6.8 (5.3, 8.8) | 7.3 (5.7, 9.4) | 0.352 | 0.191 |
| NEU (10⁹/L), median (IQR) | 4.2 (3.0, 6.0) | 4.7 (3.2, 6.7) | 0.320 | 0.219 |
| LYM (10⁹/L), median (IQR) | 1.59 (1.19, 2.03) | 1.52 (1.12, 1.97) | 0.572 | 0.112 |
| MON (10⁹/L), median (IQR) | 0.52 (0.35, 0.69) | 0.57 (0.37, 0.74) | 0.350 | 0.196 |
| RBC (10¹²/L), mean (SD) | 4.23 (0.65) | 4.16 (0.72) | 0.616 | 0.106 |
| HGB (g/L), mean (SD) | 119.22 (23.70) | 115.20 (26.20) | 0.430 | 0.167 |
| HCT (L/L), mean (SD) | 36.96 (7.00) | 35.90 (8.10) | 0.498 | 0.148 |
| PLT (10⁹/L), median (IQR) | 252 (188, 333) | 271 (201, 360) | 0.408 | 0.174 |
| INR, mean (SD) | 1.01 (0.14) | 1.02 (0.18) | 0.771 | 0.068 |
| Fbg (g/L), median (IQR) | 3.58 (2.92, 4.70) | 3.83 (3.10, 5.06) | 0.377 | 0.187 |
| D-dimer (ng/L), median (IQR) | 0.85 (0.47, 1.84) | 1.02 (0.55, 2.25) | 0.482 | 0.161 |
| Early irAE (grade ≥2) |  |  | 0.368 | 0.182 |
| Yes | 52 (30.6%) | 7 (22.6%) |  |  |
| No | 118 (69.4%) | 24 (77.4%) |  |  |

**Table note:** BMI, body mass index; irAE, immune-related adverse event; IgA, immunoglobulin A; IgG, immunoglobulin G; IgM, immunoglobulin M; CD, cluster of differentiation; ALT, alanine aminotransferase; AST, aspartate aminotransferase; γ-GGT, gamma-glutamyl transferase; LDH, lactate dehydrogenase; TBIL, total bilirubin; DBIL, direct bilirubin; IDBIL, indirect bilirubin; TP, total protein; ALB, albumin; GLOB, globulin; A/G, albumin-to-globulin ratio; PALB, prealbumin; BUN, blood urea nitrogen; CREA, creatinine; UA, uric acid; ALP, alkaline phosphatase; Glu, glucose; WBC, white blood cell count; NEU, neutrophil count; LYM, lymphocyte count; MON, monocyte count; RBC, red blood cell count; HGB, hemoglobin; HCT, hematocrit; PLT, platelet count; INR, international normalized ratio; Fbg, fibrinogen; D-dimer, fibrin degradation product D-dimer; BCLC, Barcelona Clinic Liver Cancer; IQR, interquartile range; SD, standard deviation.

**Supplementary Table 5** Complete performance metrics of the prediction models in the training and external validation cohorts

| **Model** | **Cohort** | **AUC (95% CI)** | **Sensitivity** | **Specificity** | **PPV** | **NPV** | **F1 score** | **Brier score** | **Calibration intercept** | **Calibration slope** |
| --- | --- | --- | --- | --- | --- | --- | --- | --- | --- | --- |
| TabNet | Training | 0.85 (0.81–0.89) | 78.70% | 78.50% | 62.40% | 89.10% | 69.60% | 0.158 | 0.01 | 0.97 |
| TabNet | External validation | 0.78 (0.71–0.85) | 79.70% | 66.90% | 50.00% | 88.80% | 61.40% | 0.181 | 0.03 | 0.92 |
| Random forest | Training | 0.79 (0.74–0.83) | 72.30% | 72.10% | 53.90% | 85.20% | 61.80% | 0.184 | 0.04 | 0.84 |
| Random forest | External validation | 0.73 (0.65–0.81) | 71.20% | 62.00% | 43.80% | 83.80% | 54.20% | 0.194 | 0.05 | 0.8 |
| XGBoost | Training | 0.85 (0.82–0.89) | 80.90% | 81.10% | 65.90% | 90.40% | 72.70% | 0.15 | -0.02 | 1.06 |
| XGBoost | External validation | 0.69 (0.61–0.77) | 61.00% | 65.50% | 42.40% | 80.20% | 50.00% | 0.21 | -0.1 | 0.62 |
| Support vector machine | Training | 0.77 (0.72–0.82) | 68.10% | 70.20% | 50.80% | 83.00% | 58.20% | 0.193 | 0.06 | 0.78 |
| Support vector machine | External validation | 0.71 (0.63–0.79) | 66.10% | 64.10% | 43.30% | 82.00% | 52.30% | 0.202 | 0.07 | 0.76 |
| Logistic regression | Training | 0.72 (0.67–0.77) | 63.80% | 68.60% | 47.90% | 80.80% | 54.70% | 0.204 | 0.02 | 0.88 |
| Logistic regression | External validation | 0.70 (0.62–0.78) | 61.00% | 66.20% | 42.90% | 80.30% | 50.30% | 0.207 | 0.02 | 0.84 |

**Note:** PPV, positive predictive value; NPV, negative predictive value. Threshold-based metrics were calculated using model-specific cut-offs derived from the training cohort by the Youden index and then applied to the external validation cohort without further adjustment.

**Supplementary Table 6** Bootstrap comparison of AUCs between TabNet and comparator models in the external validation cohort

| **Comparison** | **AUC of TabNet** | **AUC of comparator** | **ΔAUC** | **95% CI for ΔAUC** | **P value** |
| --- | --- | --- | --- | --- | --- |
| TabNet vs Random Forest | 0.78 | 0.73 | 0.05 | -0.015 to 0.112 | 0.132 |
| TabNet vs XGBoost | 0.78 | 0.69 | 0.09 | 0.027 to 0.154 | 0.006 |
| TabNet vs Support vector machine | 0.78 | 0.71 | 0.07 | 0.005 to 0.138 | 0.034 |
| TabNet vs Logistic regression | 0.78 | 0.7 | 0.08 | 0.014 to 0.151 | 0.018 |

**Note:** AUC differences were estimated using bootstrap resampling in the external validation cohort. ΔAUC was calculated as AUC of TabNet minus AUC of the comparator model. P values are two-sided.

**Supplementary Table 7** Bootstrap optimism-corrected internal validation of the final TabNet model

| **Metric** | **Apparent** | **Optimism** | **Corrected** |
| --- | --- | --- | --- |
| AUC | 0.849 | 0.026 | 0.823 |
| Brier score | 0.158 | −0.011 | 0.169 |
| Calibration intercept | 0.012 | −0.012 | 0.024 |
| Calibration slope | 0.973 | 0.056 | 0.917 |

**Note:** Internal validation was performed using 500 bootstrap resamples. Estimated optimism was calculated as bootstrap-sample performance minus performance in the original training cohort, and optimism-corrected performance as apparent performance minus estimated optimism. Negative values reflect the direction of the signed difference.

**Supplementary Table 8** Sensitivity analysis comparing the full-feature and reduced-feature TabNet models

| **Metric** | **Full-feature TabNet** | **Top 10 TabNet** |
| --- | --- | --- |
| Number of predictors | 51 | 10 |
| Crude events per predictor | 2.76 | 14.1 |
| External validation AUC (95% CI) | 0.78 (0.71–0.85) | 0.75 (0.67–0.79) |
| External validation Brier score | 0.181 | 0.173 |
| Calibration intercept | 0.03 | 0.03 |
| Calibration slope | 0.92 | 0.88 |

**Note:** The reduced-feature TabNet model was developed using the 10 predictors with the highest SHAP importance identified exclusively within the training cohort. AUC, area under the receiver operating characteristic curve; CI, confidence interval.
